# Comparative proteomics of human milk casein fraction collected from women of Korean and Han ethnic groups in China

**DOI:** 10.3389/fnut.2023.1078355

**Published:** 2023-01-23

**Authors:** Cuina Wang, Yingcong Lu, Jia Hu, Yang Yang, Jianjun Cheng, Shilong Jiang, Mingruo Guo

**Affiliations:** ^1^Department of Food Science, Jilin University, Changchun, China; ^2^Department of Food Science, Northeast Agricultural University, Harbin, China; ^3^R&D Center, Heilongjiang Feihe Dairy Co., Ltd., Beijing, China; ^4^Department of Nutrition and Food Sciences, College of Agriculture and Life Sciences, The University of Vermont, Burlington, VT, United States

**Keywords:** human milk, casein, DIA technique, function annotation, Korean group

## Abstract

**Introduction:**

Human breast milk provides neonates with indispensable nutrition and function. Milk protein is one of the main constituents of breast milk. Human milk profiles can be influenced by many factors.

**Methods:**

The present study aimed to investigate the difference in casein isolated from mature milk of healthy mothers of Korean and Han ethnic groups in China using data-independent acquisition (DIA) proteomics.

**Results:**

A total of 535 proteins were identified and quantified in casein fraction samples from both groups. A total of 528 proteins were annotated to 52 Gene Ontology (GO) terms, the majority (94.13%) of which were distributed in the cell and cell parts of the cellular component. Kyoto Encyclopedia of Genes and Genomes (KEGG) analysis revealed that 106 proteins were involved in 23 pathways, the greatest (36.79%) in carbohydrate metabolism. There were 39 differentially expressed proteins (DEPs)–10 upregulated and 29 downregulated–between Korean and Han milk. The GO function of blood microparticles and KEGG pathway of *Staphylococcus aureus* infection for DEPs were the most significantly enriched (*p* < 0.05). Protein-protein interaction analysis revealed a network with 23 DEPs in 47 interactions, and the fibrinogen alpha chain ranked first as the hub protein.

**Discussion:**

These data may provide useful technical guidance for the development of specific infant foods for certain populations.

## 1. Introduction

Human milk is a vital biofluid and the perfect nutrition source for infants. It can provide various beneficial effects to breastfed infants, including antimicrobial and immune-promoting effects. Breastfeeding reduces the risk of asthma (1), diabetes (2) and diarrhea (3). The excellent nutritional and functional characteristics of human milk give is attributed to the numerous components of breast milk. To date, 1500 proteins have been reported in human milk (4); they are the most complicated and multifunctional (5).

Human milk proteins are secreted proteins proteolyzed by enzymes in the mammary gland and can be divided into three main groups: casein, whey protein, and milk fat globule membrane protein. Casein content in milk depends on the lactation period—it is approximately 20% in the early stage and 45% in the late phase (6). Caseins in milk mainly exist in the form of micelles with an average size of 40–100 nm (7). Caseins have been shown to have multiple biological functions in newborn babies, especially in transporting calcium phosphate from the mother to the infants. Caseins are a family of proteins mainly composed of α-caseins (α_s1_ and α_s2_ caseins), β-caseins, and κ-caseins. With the development of proteomics technology, casein profiles have been thoroughly explored in the milk from different species, such as goats (8), donkeys (9), humans (10), cows, buffaloes, and yaks (11).

Mass spectrometry (MS)-based proteomics is a powerful and efficient tool for the in-depth analysis of complicated milk proteins (12). Owing to the advantages of high data quality, excellent quantitative accuracy, and powerful traceability, the data-independent acquisition (DIA) mode has become an ideal choice for proteomic quantitative experiments. Different from the data-dependent acquisition (DDA) style, the DIA mode can collect MS1 and MS2 results at the same time, and obtain MS2 spectra of all parent ions, breaking the inherent non-repeatability and poor limitations (13).

Owing to maternal-neonatal diseases or work pressure, human milk is not always available for infants, and formula foods have emerged in the market. Therefore, investigating the composition of human milk is of great importance in designing optimum infant foods. However, human milk is a dynamic biofluid, and its detailed composition varies depending on many factors such as the stage of nursing, diet of mother, and maternal characteristics (5). This study aimed to investigate the differences in the composition of caseins in the milk from healthy mothers of two ethnic groups in China—Korean and Han—using the DIA method. The Han is the largest ethnic group in China, and Koreans are mainly settled in the Yanbian Korean Autonomous Prefecture, Jilin Province. A comprehensive insight into milk protein differences may provide useful technical guidance for the development of specific infant foods for certain populations.

## 2. Materials and methods

### 2.1. Sample collection

Breast milk collection was approved by the ethics committee of the Northeast Agricultural University. Eighteen women volunteers from each ethnic group (a total of 36) were recruited from the maternity care center in Yanbian Autonomous Prefecture, Jilin Province, and informed consent was obtained from all donors. All mothers received similar diets, according to the results of the questionnaire. The breastmilk samples were collected from mothers with full-term babies in the lactation stage from 14 to 28 days post-partum. The age of mothers ranged from 20 to 35 and feed babies with breastmilk. The parity of most mothers is 1 or 2. At a specific time between 9 a.m. and 11 a.m., milk was collected using an automatic breast pump. The first few drops were discarded and the collected milk samples were immediately shipped to the laboratory on dry ice. The samples were stored at −80°C until analysis. Six samples were randomly classified into one group, and three biological duplications were obtained.

### 2.2. Extraction of casein from whole milk

Proteins were extracted from human milk as previously described (14). First, human milk samples were centrifuged to obtain two layers. The skimmed layer was further separated by adjusting the pH to 4.6, where the casein fraction could be precipitated. The casein precipitate was collected, washed using precooled acetone, and then dried in a fume hood, and dissolved using 4% SDS. Bicinchoninic acid was used to quantitatively analyze casein extracted from the samples.

### 2.3. Filter-aided sample preparation (FASP) enzymolysis

Enzymatic hydrolysis of protein samples was conducted using FASP according to a previous study (15), which seems to be the most effective approach for biological sample aliquots owing to the low miscleavage rate (16). To each protein sample (100 μg) 8 M urea was added to obtain a final volume of 200 μL, and then DL-dithiothreitol was added until a final concentration of 10 mM; this mixture was incubated at 56°C for 30 min to destroy the intramolecular and intermolecular disulfide bonds. Next, iodoacetamide (IAA) at a concentration of 50 mM was used as an alkylation reagent for cysteine and histidine to ensure complete denaturation and reduction of protein samples, and the reaction mix was incubated in the dark for 40 min. The samples were transferred to a 10 K ultrafiltration tube, centrifuged at 12, 000 × *g* at room temperature, and the filtrate was discarded. Subsequently, 400 μL of urea (8 M) was centrifuged thrice at 12,000 × *g* at room temperature. Ammonium bicarbonate solution (50 mM, 200 μL) was introduced into an ultrafiltration tube and centrifuged at 12,000 × *g* at room temperature, and the filtrate was discarded. This step was repeated three times. Trypsin was placed in an ultrafiltration tube at a sample/enzyme ratio of 50/1 (mass ratio), and enzymolysis was performed at 37°C for 16 h. After enzymolysis, the digesta was centrifuged at 4°C at 12000 × *g*, and the filtrate was collected and lyophilized.

### 2.4. High performance liquid chromatography (HPLC) separation

High performance liquid chromatography (HPLC) separation of the enzymatically hydrolyzed samples was conducted using an HPLC system (Agilent 1100, USA) equipped with Waters X Bridge C18 (5 μm, 4.6 mm × 250 mm, 120 Å). This HPLC was preparative and was used to divide the peptides into 10 fractions based on polarity. The mobile phase was composed of A (98% ddH_2_O, 2% acetonitrile, pH 10) and B (98% acetonitrile, 2% ddH_2_O, pH10). The 60-min chromatographic gradient for separation was as follows: 0–5 min/97% A/0.4 mL/min, and then the flow rate was kept at 0.7 mL/min and the mobile phase change was 5–5.10 min/97% A, 5.10–10 min/95% A, 10–35 min/82% A, 35–45 min/66% A, 45–58 min/5% A, 58–60 min/97% A. Samples were collected every 1 minute.

### 2.5. Qualitative characterization of casein using DDA

The 10 fractions of peptide samples were mixed into 3 mixtures and then subjected to HPLC-MS (Thermo Scientific EASY-nLC, USA). The 60 min gradient for separation was: 0 min/3% B, 0–2 min/8% B, 2–46 min/28% B, 46–55 min/50% B, 55–56 min/100% B, 56–60 min/100% B. The flow rate was maintained at 0.6 mL/min. The MS parameters were as follows: (1) MSn1: scan range 375–1500 m/z; maximum injection time (MIT) 50 ms; AGC target 400,000; orbitrap resolution 120,000. (2) MSn2: MIT 22 ms; AGC target 50,000; orbitrap resolution 15,000; collision energy 30%.

Proteins in the milk casein fraction were qualitatively obtained using DDA. To enhance the signal of low-abundance proteins in the MS spectra, the digested peptides from each sample were mixed. Tryptic digestion was detected by LC-MS/MS using a Thermo Scientific EASY-nLC mass spectrometer. The proteins were searched using Proteome Discoverer 2.1.0182 (Thermo Fisher Scientific, Rockford, IL, USA) with Sequest HT. The data bank used was UniProt TaxId:9606. Enzyme: trypsin; miss cleavage: 2; peptide mass tolerance: ± 10 ppm; fragment mass tolerance: ± 0.02 Da; peptide false discovery rate (FDR): less than 1%.

### 2.6. DIA data analysis

The samples were then subjected to HPLC-MS (Thermo Scientific Orbitrap Fusion Lumos, USA). The gradient for separation and flow rate was consistent with those of the DDA described above. The DIA MS parameters were as follows: (1) MSn1: scan range 350–1,500 m/z; MIT 50 ms; AGC target 400,000; Orbitrap resolution 60,000. (2) MSn2: scan range 200–2,000 m/z; MIT 54 ms; AGC target 300,000; orbitrap resolution 30,000; collision energy 33%.

DIA was used for quantitative analysis of proteins using Skyline software (Department of Genome Sciences, University of Washington, Ave. NE, Seattle, WA). The relative quantitation of proteins was performed using the normalized spectral count method. The parameters were set as follows: sub-ion M/Z: larger than parent ion and last ion-3; maximum number of sub-ions: 5; minimum number of sub-ions: 2; sub-ion extraction window: 5 min; dotp: greater than or equal to 0.6.

### 2.7. Determination of differentially expressed proteins (DEPs)

The significance level, expressed as p values, was estimated using the t-test. DEPs were obtained using standards of fold change (FC) > 1.5 or < 0.67 and p < 0.05. Benjamin-Hochberg analysis was employed to obtain the corrected p values (q value) for GO and KEGG enrichment analysis.

### 2.8. Biological information analysis

All identified proteins were analyzed for annotation in terms of Gene Ontology (GO) and Kyoto Encyclopedia of Genes and Genomes (KEGG) analyses using DAVID Bioinformatics Resources 6.8 (http://david.abcc.ncifcrf.gov/home.jsp). Proteins differentially expressed between the two groups were subjected to GO and KEGG enrichment analyses. Protein-protein interaction (PPI) networks between differentially expressed proteins for each comparison were examined using the STRING online database (http://string-db.org) with a minimum required interaction score of 0.4, and figures were drawn using Cytoscape (Version 3.7.1). The Cytohubba plug-in was used to analyze the top 10 hub proteins using the MCC method.

## 3. Results and discussion

### 3.1. Identified caseins from the studied human milk groups

Proteomic investigation of the human milk casein fraction by qualitative DDA mode (567 proteins) and quantitative DIA technology revealed a total of 535 proteins (Supplementary Table 1 listed the detailed information). Among all the identified proteins, major caseins, such as β-casein (W5RWE1), κ-casein (P07498), and α_s1_-casein (E9PDQ1), were observed, with β-casein having the highest relative abundance, followed by κ-casein and α_s1_-casein. β-casein consists of 226 amino acids and is highly phosphorylated (10). Recently, β-casein has been developed as a natural nanocarrier to encapsulate and deliver hydrophobic nutrients to enhance their bioavailability (17). κ-casein has a highly glycosylated C-terminus and exhibits both antibacterial and prebiotic effects. *In vitro* studies have shown that κ-casein can inhibit the binding of *Helicobacter pylori* to human gastric mucosa while boosting the proliferation of *Bifidobacterium infantis* and *Lactobacillus bifidus* (18). α_s1_-Casein contains cysteine, which can form disulfide links with κ-casein, and the low degree of phosphorylation benefits the infant immune system (19). As shown in Supplementary Table 1, lysozyme (P61626), the main whey protein, was also detected in the casein fraction. As indicated in a recent study, approximately 75% of lysozyme in human milk is naturally bound to casein, and this association does not affect the antibacterial activity of lysozyme (7).

### 3.2. Function annotation of caseins quantified in human milk

**FIGURE 1:**
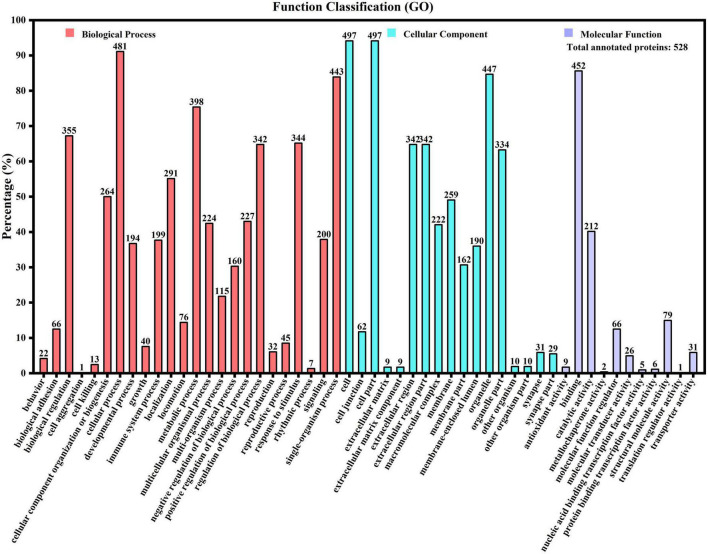
Gene Ontology function annotation of human milk caseins from Korean and Han ethnic groups in China.

To reveal the potential physiological function of casein, all identified and relatively quantified proteins were annotated using the GO, KEGG, and EggNOG databases. GO is a standardized system for gene function classification. Figure 1 shows that 528 proteins were annotated with 52 terms, which were further clustered into 24, 17, and 11 terms in categories of biological process (BP), cellular component (CC), and molecular function (MF), respectively. In each category, the terms with the greatest number of proteins were cellular process (481 proteins, 91.1%), cell, cell part (497 proteins, 94.13%), and binding (452 proteins, 85.61%) for BP, CC, and MF, respectively. The most prevalent molecular function of the binding activity has been reported in other studies (11). From the perspective of protein types involved in GO function annotation, it was found that the most functional protein involved in the greatest number of GO function items was the amyloid-beta A4 protein (A0A140VJC8). This protein acts as a cell-surface receptor and has roles in axon growth, neuronal adhesion, and axon formation on the surface of neurons (20).

**FIGURE 2:**
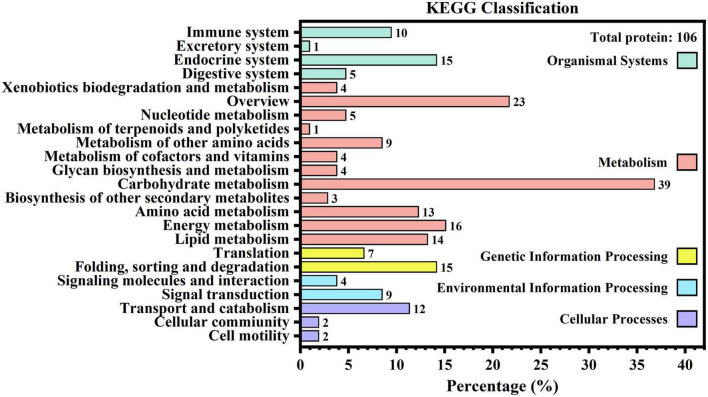
Kyoto Encyclopedia of Genes and Genomes metabolic pathway of human milk caseins from Korean and Han ethnic groups in China.

KEGG results can be interpreted from the following five categories: cellular processes, environmental information processing, genetic information processing, metabolism, and organismal systems. Figure 2 shows that only 106 proteins were found to be related to 23 secondary branches, which fell into 3, 2, 2, 12, and 4 pathways for the aforementioned five categories, respectively. Within each branch, the specific pathways with the greatest number of proteins were transport and catabolism (12, 11.32%), signal transduction (9, 8.49%), folding, sorting, and degradation (15, 14.15%), carbohydrate metabolism (39, 36.79%), and the endocrine system (15, 14.15%). It was also found that aldehyde dehydrogenase (NAD(P) +) (A0A1B0GW77) was involved in most KEGG pathways, up to 14.

**FIGURE 3:**
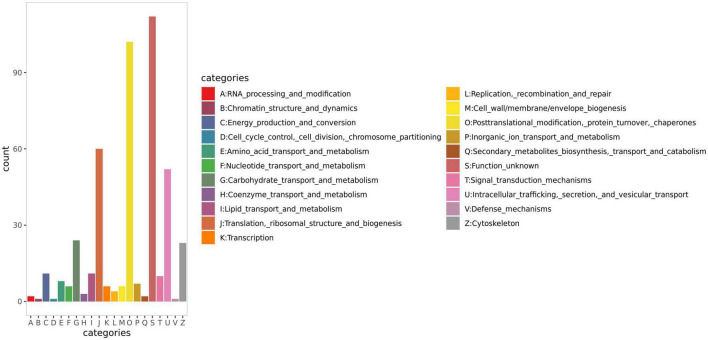
EggNOG annotation result of human milk caseins from Korean and Han ethnic groups in China.

Functional classification of all the identified proteins was performed using EggNOG. The annotation results (Figure 3) indicated that, besides unknown functions (S), ‘post-translational modification, protein turnover, chaperone’ (D) were the most enriched functional class, with a total of 102 proteins, followed by ‘translation, ribosomal structure, biogenesis’ (J), and ‘intracellular trafficking, secretion, vesicular transport’ (U) with 60 and 52 proteins, respectively.

### 3.3. Comparative analysis of DEPs

As shown in Table 1, 39 proteins, representing approximately 7.3% of the total proteins, were significantly differentially (*p* < 0.05) expressed between the two groups. The 39 DEPs between Korean and Han may be related to the differences in ethnicity caused by genetic variation. Han ethics group is the group with the largest population of about 1.2 billion, and there are 55 ethnic minorities. Korean ethics group in the northeast of China has obvious characteristics and has a population of about 2 million. In this study, we studied the protein differences between the milk samples of Han and Korean ethics groups in the northeast of China (Jilin Province). As far as we know, this is the first study regarding the investigation of protein differences in human milks between the Han and Korean ethics groups. However, differences in human milk serum from different ethics groups which are distributed in Yunnan (Han and Bai ethnicity), Gansu (Han and Tibetan ethnicity), Xinjiang (Uygur ethnicity), and Inner Mongolia (Mongolian ethnicity) were studied in a previous study (21) and the authors found that 34 proteins significantly differed with geography and ethnicity. Our results were similar with this study. Although the women from different groups had a similar diet during the first month after giving birth to a child since they all live in the care center. However, there was a large difference in the traditional diet for the two ethics groups. Therefore, traditional diet may be another reason for the differences in proteins between these two groups. Additionally, the life style of people in Han and Korean ethics groups was different in terms of culture, behavioral habits, socioeconomic and environmental settings, which may be other influencing factors for the differences (39). Eight immunity-related proteins (PIGR, IGK, immunoglobulin J chain, immunoglobulin alpha-2 heavy chain, and four immunoglobulin fragments) were included in the DEPs, indicating their possible role in the critical phase of immunological immaturity of the newborn. It can be concluded that the abundance of immune-related proteins in milk produced by mothers depends on the mother’s life circumstances, which may be related with ethnicity and diet (21). PIGR is the polymeric Ig receptor when IgA and IgM are transported across epithelial cells into milk, and the polypeptide of the joining (J) chain provides antibodies with the capacity to bind PIGR (22). The five differentially expressed enzymes, tyrosine 3-monooxygenase/tryptophan 5-monooxygenase activation protein, cathepsin S, mannosyl-oligosaccharide 1,2-alpha-mannosidase IA, asparaginyl endopeptidase, and aldehyde dehydrogenase NAD (+), indicated a possible difference in human milk casein fractions in the processes of glycolipid metabolism, protein degradation, carbohydrate hydrolysis, and the removal of excess aldehydes in the body.

**TABLE 1:** Differentially expressed proteins in human milk casein (Korean VS. Han).

ID	Name	FC	*P*-value	Significance
A0A024R035	Complement component C9	5.743789	0.001342	Up
A0A1W2PRS1	Lysosome membrane protein 2 (Fragment)	4.339686	0.012025	Up
A0A1B0GW77	Aldehyde dehydrogenase (NAD(+))	2.871715	0.034486	Up
P33908	Mannosyl-oligosaccharide 1,2-alpha-mannosidase IA	2.792878	0.035985	Up
A8K690	cDNA FLJ76863, highly similar to Homo sapiens stress-induced-phosphoprotein 1 (Hsp70/Hsp90-organizing protein) (STIP1), mRNA	2.441516	0.016106	Up
A0A024R1S8	LIM and SH3 domain protein 1	2.262799	0.016762	Up
J3QQX2	Rho GDP-dissociation inhibitor 1	2.004956	0.015161	Up
A0A024R240	Epididymis secretory sperm binding protein	1.828674	0.0475	Up
A0A0C9WPF5	Unplaced genomic scaffold K443scaffold_349, whole genome shotgun sequence	1.73154	0.036606	Up
Q6FG99	RPLP1 protein	1.716638	0.010578	Up
C9JC84	Fibrinogen gamma chain	0.637988	0.036707	Down
A0A024R1K7	Tyrosine 3-monooxygenase/tryptophan 5-monooxygenase activation protein, eta polypeptide, isoform CRA_b	0.634994	0.036992	Down
B2R7F8	Plasminogen	0.626325	0.021449	Down
P01833	Polymeric immunoglobulin receptor	0.61087	0.031114	Down
Q14444	Caprin-1	0.570919	0.034254	Down
P25774	Cathepsin S	0.568235	0.048096	Down
P80303	Nucleobindin-2	0.565948	0.00541	Down
A0A5C2GJR5	IG c837_light_IGKV1-39_IGKJ2 (Fragment)	0.565674	0.022649	Down
Q59EJ3	Heat shock 70kDa protein 1A variant (Fragment)	0.559644	0.029218	Down
P01591	Immunoglobulin J chain	0.550935	0.007248	Down
P02671	Fibrinogen alpha chain	0.54075	0.012335	Down
Q6P5S8	IGK@ protein	0.532154	0.003905	Down
Q6ZP37	cDNA FLJ26554 fis, clone LNF01773, highly similar to Galactokinase	0.518555	0.026725	Down
A0A5C2G585	IGL c1483_light_IGKV1-5_IGKJ2 (Fragment)	0.508055	0.033971	Down
P01024	Complement C3	0.497846	0.04343	Down
P13987	CD59 glycoprotein	0.47941	0.013143	Down
A0A5C2GLT5	IG c1145_heavy_IGHV1-18_IGHD3-22_IGHJ6 (Fragment)	0.471334	0.02293	Down
A0A7S5EXD1	IGH c1503_heavy_IGHV3-49_IGHD1-1_IGHJ6 (Fragment)	0.450834	0.016916	Down
Q05D08	PA2G4 protein (Fragment)	0.445366	0.030757	Down
O00468	Agrin	0.435279	0.048526	Down
P08962	CD63 antigen	0.425141	0.01487	Down
A8K7Q1	Nucleobindin-1	0.390836	0.000159	Down
B3KS79	cDNA FLJ35730 fis, clone TESTI2003131, highly similar to ALPHA-1-ANTICHYMOTRYPSIN	0.390438	0.037523	Down
P0DOX2	Immunoglobulin alpha-2 heavy chain	0.366337	0.038222	Down
A2J1N5	Rheumatoid factor RF-ET6 (Fragment)	0.346707	0.0254	Down
O43242	26S proteasome non-ATPase regulatory subunit 3	0.345703	0.034891	Down
P01023	Alpha-2-macroglobulin	0.333425	0.030029	Down
A8K669	Asparaginyl endopeptidase	0.288391	0.039519	Down
P02787	Serotransferrin	0.18595	0.004538	Down

**FIGURE 4:**
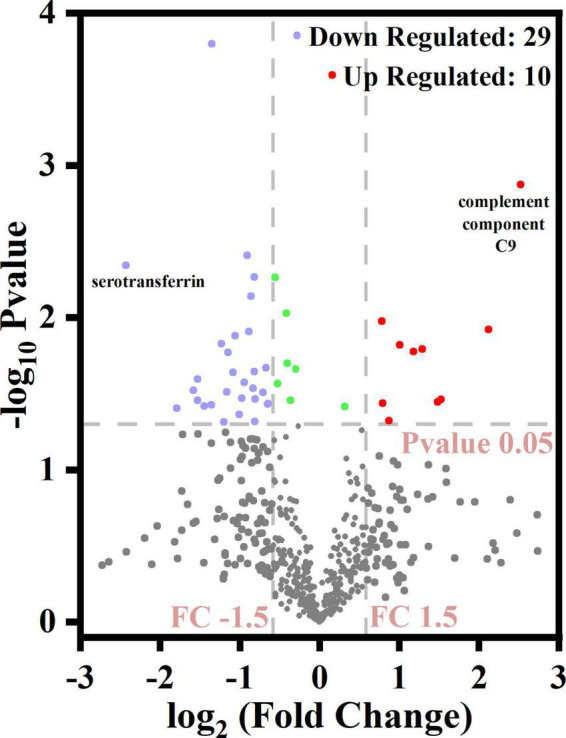
Volcano diagram for differentially expressed caseins from Korean and Han ethnic groups in China.

**FIGURE 5:**
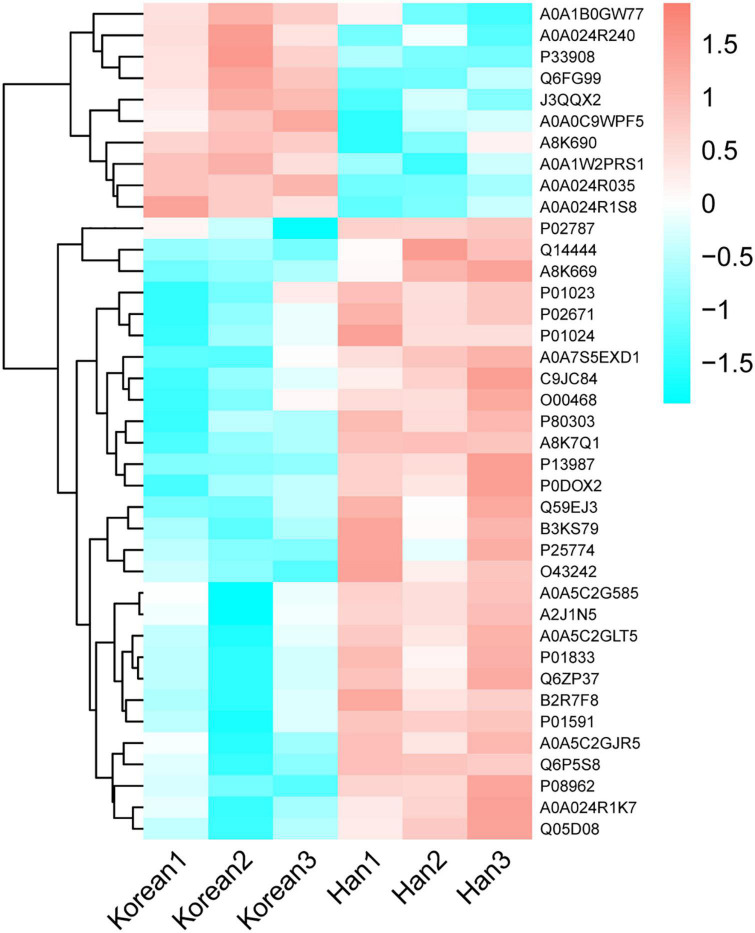
Hierarchical clustering of differentially expressed caseins from Korean and Han ethnic groups in China. Each column is a group. Each row is a protein. Red part represents a significantly upregulated protein, cyan part represents a significantly downregulated protein, and white part represents a protein that does not change significantly.

The volcano plot (Figure 4) revealed that 10 DEPs were upregulated (red color) and 29 were downregulated (blue color) among the differentially expressed proteins. Figure 5 shows the hierarchical clustering of 39 DEPs with three biological replicates in each group, which displays the upregulated and downregulated proteins. Hierarchical clustering can be used to group proteins with similar structures, which helps investigate protein functions. For example, nucleobindin-1 (A8K7Q1) and nucleobindin-2 (P80303) are divided into one group, and research has shown that they are homologous multidomain calcium and DNA binding proteins (23) and have multiple functions, including regulation of inflammation and bone formation (24).

Among the upregulated proteins, complement component C9 (A0A024R035) had the highest difference (the highest FC value) between the two groups with a log2FC of 2.52, *P* = 0.0013. As a single-chain glycoprotein, C9 is the final product of the complement cascade, which is part of the membrane attack complex (MAC). C9 can adhere to the cell surface, thus destroying the integrity of the cell membrane and resulting in osmotic lysis and cell death (25). Among the downregulated proteins, serotransferrin (P02787), an iron-binding glycoprotein, had the lowest log2FC value of −2.43, *p* = 0.0045. In addition to regulating the iron content of biological fluids, serotransferrin has been associated with a variety of diseases such as atransferrinemia and cardiovascular diseases. The properties of serotransferrin and its receptor can be exploited to deliver drugs specifically into the brain and cancer cells (26).

### 3.4. Enrichment analysis of GO functions for DEPs

**FIGURE 6:**
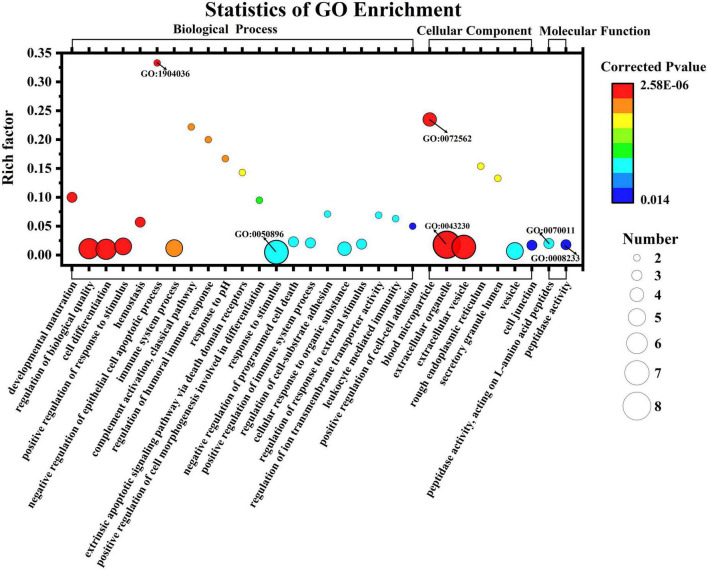
Gene Ontology enrichment classification of differentially expressed casein from Korean and Han ethnic groups in China.

**FIGURE 7:**
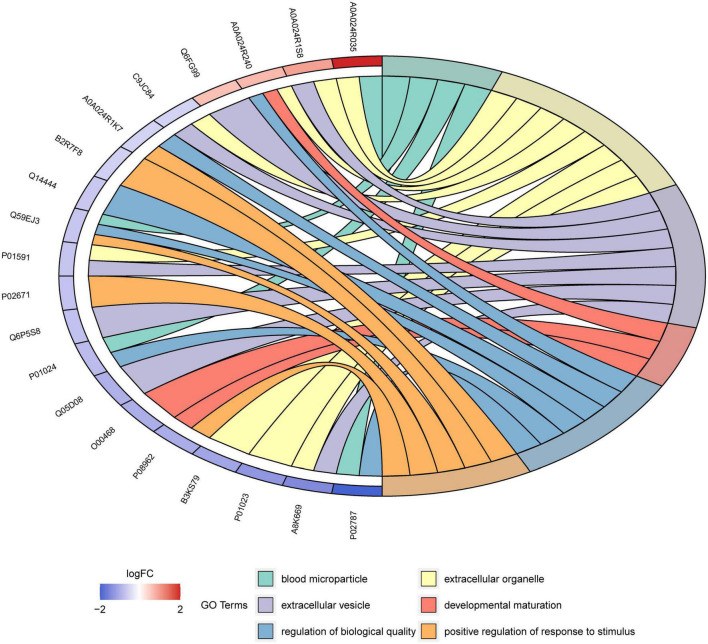
Chord diagram for the first six enriched GO terms from Korean and Han ethnic groups in China.

Enrichment analysis revealed that 125 of the 545 GO terms in which DEPs participated were significantly enriched (*q* < 0.05). The top 30 enriched GO terms are displayed in the bubble chart in Figure 6. The functions with the most proteins were response to stimulus (7 proteins, GO:0050896), extracellular organelle (8, GO:0043230), and peptidase activity, acting on L-amino acid peptides (3, GO:0070011) and peptidase activity (3, GO:0008233) for BP, CC, and MF categories, respectively. This means most of the DEPs were related with these functions and the human milk from Korean and Han exhibited different functions toward infants due to the differences in the proteins. These differences may be due to genetic background, lifestyle, and environmental factors. Blood microparticles (4, GO: 0072562) were the most significant GO terms, with a rich factor of 0.235 and a minimum *q* value of 2.58E-06. Among all the significantly enriched terms, negative regulation of the empirical cell apoptotic process (2, GO: 1904036) had the highest rich factor of 0.33. Figure 7 shows the DEPs involved in the above GO terms in the form of a chord diagram; it is obvious that negative regulation of the empirical cell apoptotic process included the fibrinogen alpha chain and fibrinogen gamma chain. As an abundant plasma glycoprotein, fibrinogen consists of two copies of chains: Aα, Bβ, and γ (27). As a critical part of hemostasis, water-soluble fibrinogen forms a network of fibrin fibers with the participation of thrombin and coagulates into blood clots (28). Besides participating in blood coagulation, fibrinogen is involved in inflammation, cell migration, and tumorigenesis (27). Moreover, the immunoglobulin J chain and the two immunoglobulin fragments are both involved in the response to the stimulus. C3, C9, and serotransferrin are present in blood microparticles. There are two enzymes in the chord diagram, cathepsin S and asparaginyl endopeptidase, both of which participate in peptidase activity, and the latter also participates in extracellular organelle and peptidase activity, acting on L-amino acid peptides.

### 3.5. Enrichment analysis of KEGG pathways for DEPs

**FIGURE 8:**
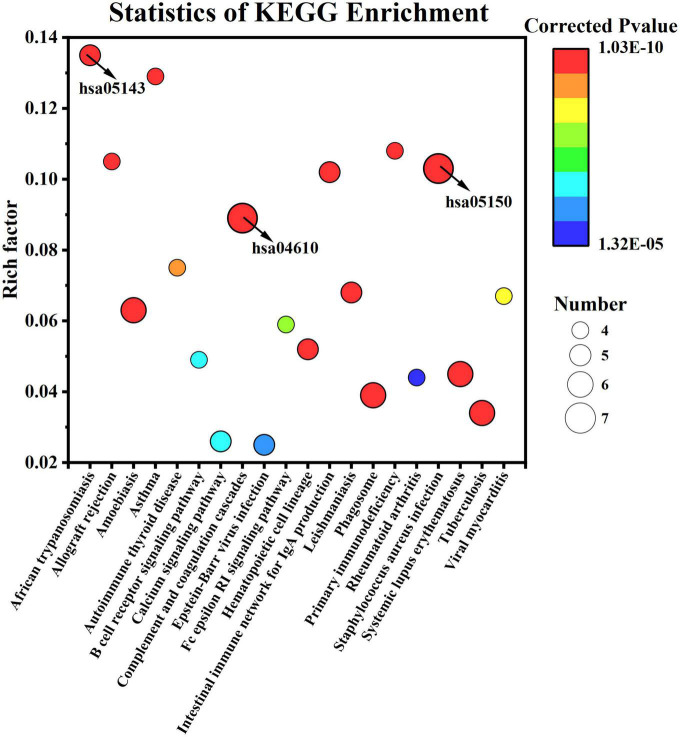
Kyoto Encyclopedia of Genes and Genomes pathway enrichment of differentially expressed caseins from Korean and Han ethnic groups in China.

For KEGG enrichment analysis, 35 of the 113 KEGG pathways were significantly enriched (q < 0.05); the first 20 pathways are displayed in Figure 8. Having a rich factor of 0.103, *Staphylococcus aureus* infection (hsa05150) was the most significant pathway with a minimum q value of 1.03E-10. In terms of rich factors, African trypanosomiasis (5, hsa05143) had a maximum value of 0.135. As for the pathway with the largest number of proteins, *S. aureus* infection and complement and coagulation cascades (0.089, 1.39E-10, hsa04610) involved seven proteins.

As showed above, complement and coagulation cascades pathway possessed the largest number of proteins, meaning that the human milk samples between the two groups may exhibit different effects on infants in term of improving immune system. As discussed in Section 3.3, eight immunity-related proteins were included in the DEPs, which may be the reason. Composed of more than 30 proteins, the complement system participates in both innate and adaptive immunity (29). As a vital part of multiple pathways of the immune system, the complement system not only participates in host defense but also involves many physiological systems, such as the coagulation cascade (30). The complement system can be activated through three different pathways-the classical, alternative, and lectin pathways—and the 3 pathways merge at C3 (31). The complement cascade reaction rapidly mobilizes complement system proteins in response to infection or tissue damage and ends with the formation of MAC and C5a (30). MAC has been extensively studied for its ability to be inserted into cell membranes to induce cell lysis (30).

The coagulation cascade starts with primary hemostasis and then releases coagulation factors for secondary hemostasis. At this time, internal and external pathways activate factor X. Finally, activated factor X cleaves prothrombin into thrombin, converting fibrinogen into fibrin, and initiating fibrin polymerization at the site of *injury* (32). *As mentioned above, parts of the complement system (C3, C9), fibrinogen (fibrinogen alpha and* gamma chain) with coagulation ability, and plasminogen involved in wound healing are involved in this pathway. The other proteins involved in this pathway are α-2-macroglobulin and CD59 glycoprotein.

### 3.6. Pathway of *Staphylococcus aureus* infection

**FIGURE 9:**
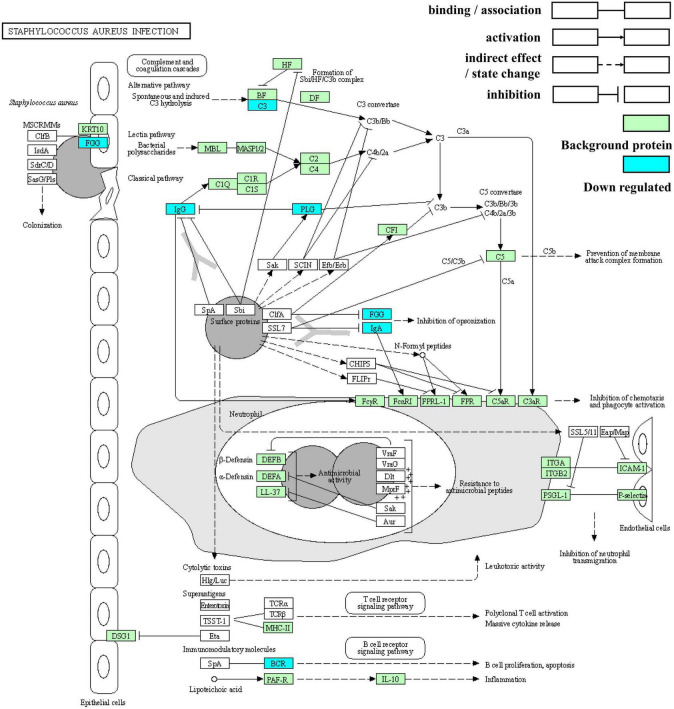
Kyoto Encyclopedia of Genes and Genomes enrichment pathway map of *Staphylococcus aureus* infection.

*Staphylococcus aureus* has attracted much attention as an important pathogen in infant infections. Newborns are likely to be infected with *S. aureus* through the birth canal, breastfeeding, and contact with people and the surrounding environment, thus increasing the possibility of short-term morbidity and mortality and adverse long-term outcomes (33). Based on the above results, *S. aureus* infection was the most significantly enriched in both ethnic groups and possessed the greatest number of DEPs. It is thus, worth exploring and its pathway map is shown in Figure 9.

Clumping factor B (ClfB) with the ability to bind to fibrinogen can adhere to human cytokeratin 10. *S. aureus* on entry into the human body stimulates three different pathways in the complement system to produce chemoattractants that can cause neutrophils to move to the infected area. In response to the immune response, *S. aureus* also has multiple mechanisms that interfere with complement through the inactivation or isolation of key components. First, cell wall-associated protein A (SpA) binds to IgG Fc fragments to prevent phagocytosis and classical pathway complement fixation (34). SpA also acts as a B-cell superantigen through interactions with the heavy chain variable part of the Fab fragments. Sbi domains III and IV interact with complement factor C3 to interfere with all three complement pathways (35). Additionally, staphylokinase can activate plasminogen (PLG), thereby cleaving the surface-bound C3b and IgG and reducing the phagocytosis of neutrophils. Moreover, clumping factor A (ClfA) can bind to fibrinogen, resulting in the enhanced degradation of C3b. Finally, the secreted superantigen-like protein (SSL7) binds to IgA, thus hindering phagocytosis (34).

Two immunoglobulin fragments (A0A5C2GLT5 and A0A7S5EXD1), immunoglobulin alpha-2 heavy chain, plasminogen, fibrinogen gamma chain, C3, and rheumatoid factor RF-ET6 fragment (A2J1N5) are the proteins involved in *S. aureus* infection. As the precursor of plasmin, plasminogen is encoded by the *PLG* gene on chromosome band 6q26 (36) and is composed of five kringle-like domains containing “lysine-binding sites” and a C-terminal domain homologous to other trypsin-like proteases (37). In addition to its significant fibrinolysis, plasminogen triggers other enzymatic cascades, including complement. Moreover, it affects immune and inflammatory processes by binding to specific cell surface receptors (plasminogen, an enigmatic zymogen).

### 3.7. PPI network analysis formed by DEPs

**FIGURE 10:**
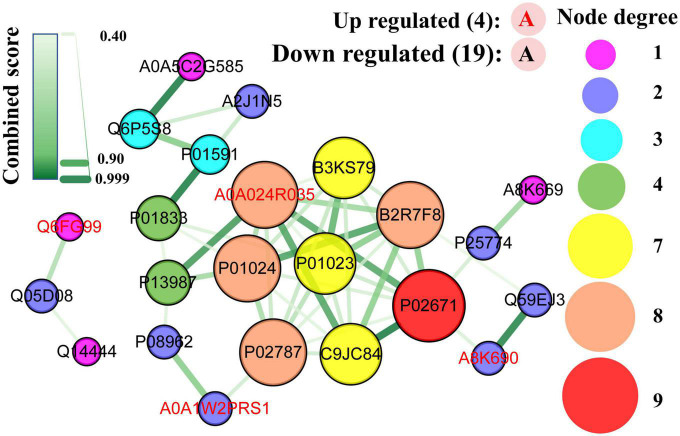
Protein-protein interaction network map of differentially expressed caseins from Korean and Han ethnic groups in China. Each node represents a protein, and each edge represents the direct interaction between proteins.

All DEPs formed a network (Figure 10) with 23 nodes and 47 edges (combined score > 0.4), indicating that 23 DEPs (19 downregulated and 4 upregulated) interacted with one another through 47 interactions. The hub proteins in the network were assessed using the MCC method, and the following proteins were ranked as the top 10: fibrinogen alpha chain (1,442), C9 (1,442), plasminogen (1,441), serotransferrin (1,441), fibrinogen gamma chain (1,440), cDNA FLJ35730 fis, highly similar to α-1-antichymotrypsin (B3KS79, 1440), C3 (724), α-2-macroglobulin (721), CD59 glycoprotein (5), and PIGR (4).

As mentioned earlier, the fibrinogen alpha and gamma chains were involved in the GO term of negative regulation of the empirical cell apoptotic process, which had the largest rich factor. As part of MAC (38), C9 was the most upregulated protein, whereas serotransferrin had the lowest FC value. The polymeric immunoglobulin receptor was the protein with the highest abundance in DEPs. As discussed in the KEGG enrichment analysis, the complement and coagulation cascades are the pathways with the most DEPs, and it can be observed that seven out of the ten hub proteins, namely, fibrinogen alpha chain, gamma chain, C9, plasminogen, C3, alpha-2-macroglobulin, and CD59 glycoprotein were obtained from this pathway. Additionally, fibrinogen gamma chain, plasminogen, and C3 were enriched in *Staphylococcus aureus* infection, which had the lowest significance level and the most proteins as discussed in the enrichment analysis.

## 4. Conclusion

DIA-based quantitative proteomics was used to investigate the differences in human milk casein fractions collected from Korean and Han women in China. A total of 535 proteins were identified, and approximately 7.2% (38) were significantly differentially expressed across the two ethnic groups. These DEPs were associated with 125 and 35 significantly enriched GO terms and KEGG pathways, respectively. Most DEPs fell into the term of response to stimulus, extracellular organelle, peptidase activity, acting on L-amino acid peptides/peptidase activity for BP, CC, and MF categories. And most DEPs were related with pathways of *S. aureus* infection and complement and coagulation cascades. These proteins interacted with each other to form a network of 23 proteins in 47 interactions. Comprehensive estimation of casein composition and function in human milk of lactating mothers from different ethnic groups aids in developing infant formulas for babies of different ethnic groups.

## Data availability statement

The datasets presented in this study can be found in online repositories. The names of the repository/repositories and accession number(s) can be found in the article/Supplementary material.

## Author contributions

CW and YL: conceptualization, investigation, formal analysis, visualization, validation, and writing—review and editing. JH: software and data curation. YY, JC, and SJ: investigation, formal analysis, and visualization. MG: conceptualization, investigation, validation, writing—original draft, writing—review and editing, and funding acquisition. All authors contributed to the article and approved the submitted version.
