# Supplementary material for: Comparative proteomics of human milk casein fraction collected from women of Korean and Han ethnic groups in China
Source: Front Nutr. 2023 Jan 23;10:1078355. doi: 10.3389/fnut.2023.1078355 (PMC9901581; doi:10.3389/fnut.2023.1078355)
Supplement: Supplementary file 1 [file Table_1.docx]

Table S1 All proteins identified in human milk casein.

| **ID** | **Name** | **FC (Korean VS. Han)** | **p value** |
| --- | --- | --- | --- |
| **A0A024QYR8** | **Transmembrane 9 superfamily member** | **0.301547946** | **0.137969958** |
| **A0A024QZ95** | **Olfactomedin 4, isoform CRA_a** | **0.924721532** | **0.887245451** |
| **A0A024R035** | **Complement component C9** | **5.743788706** | **0.001342086** |
| **A0A024R1A3** | **Ubiquitin-activating enzyme E1** | **0.291326255** | **0.38360956** |
| **A0A024R1F4** | **HCG2010666, isoform CRA_a** | **1.10194624** | **0.525418384** |
| **A0A024R1K7** | **Tyrosine 3-monooxygenase/tryptophan 5-monooxygenase activation protein, eta polypeptide, isoform CRA_b** | **0.634993954** | **0.036992312** |
| **A0A024R1S8** | **LIM and SH3 domain protein 1** | **2.262799086** | **0.016761745** |
| **A0A024R1U4** | **RAB5C, member RAS oncogene family, isoform CRA_a** | **1.060161252** | **0.669673952** |
| **A0A024R1Z6** | **Vesicle amine transport protein 1 homolog (T californica), isoform CRA_a** | **0.590018705** | **0.222301868** |
| **A0A024R240** | **Epididymis secretory sperm binding protein** | **1.828673635** | **0.047499566** |
| **A0A024R321** | **Filamin B, beta (Actin binding protein 278), isoform CRA_a** | **16.03119694** | **0.114839211** |
| **A0A024R324** | **Epididymis secretory sperm binding protein** | **1.106235167** | **0.468074432** |
| **A0A024R3Q0** | **ADP-ribosylation factor 1, isoform CRA_a** | **0.655264342** | **0.330844252** |
| **A0A024R4M0** | **40S ribosomal protein S9** | **1.362637595** | **0.473952405** |
| **A0A024R5H1** | **Folate receptor 1 (Adult), isoform CRA_a** | **0.743170403** | **0.196329754** |
| **A0A024R5K1** | **Coronin** | **2.144058923** | **0.1826361** |
| **A0A024R5Z9** | **Pyruvate kinase** | **0.566749364** | **0.072754355** |
| **A0A024R694** | **Actinin, alpha 1, isoform CRA_a** | **1.399055042** | **0.368259585** |
| **A0A024R6K8** | **T1-TrpRS** | **1.402321866** | **0.435587297** |
| **A0A024R6U7** | **ADGRG1 C-terminal fragment** | **0.621181689** | **0.342822408** |
| **A0A024R718** | **Nicotinamide phosphoribosyltransferase** | **1.218498618** | **0.428794833** |
| **A0A024R7V6** | **RAB2, member RAS oncogene family, isoform CRA_a** | **3.001881501** | **0.098473157** |
| **A0A024R814** | **Ribosomal protein L7, isoform CRA_a** | **1.315154088** | **0.50371402** |
| **A0A024R821** | **Eukaryotic translation initiation factor 3 subunit B** | **0.301441815** | **0.250043151** |
| **A0A024R882** | **Stomatin, isoform CRA_a** | **0.942508118** | **0.907022427** |
| **A0A024R969** | **Chitinase-3-like protein 1** | **0.870488805** | **0.650444213** |
| **A0A024R9D3** | **60S ribosomal protein L30** | **2.578583422** | **0.092679335** |
| **A0A024R9Q1** | **Thrombospondin 1, isoform CRA_a** | **1.561388749** | **0.284496991** |
| **A0A024RAD5** | **Dolichyl-diphosphooligosaccharide--protein glycosyltransferase 48 kDa subunit** | **0.926243209** | **0.883665637** |
| **A0A024RAR8** | **Aminopeptidase** | **1.95551449** | **0.150139018** |
| **A0A024RC65** | **HCG1991735, isoform CRA_a** | **0.796343669** | **0.159252667** |
| **A0A024RCN6** | **Valyl-tRNA synthetase** | **1.242595708** | **0.210300781** |
| **A0A024RD80** | **Heat shock protein 90kDa alpha (Cytosolic), class B member 1, isoform CRA_a** | **0.74954186** | **0.182903852** |
| **A0A024RDE6** | **Secreted phosphoprotein 1 (Osteopontin, bone sialoprotein I, early T-lymphocyte activation 1), isoform CRA_c** | **0.892891032** | **0.320879107** |
| **A0A024RDE8** | **PDZ and LIM domain 5, isoform CRA_c** | **0.688589274** | **0.434182517** |
| **A0A024RDL1** | **Chaperonin containing TCP1, subunit 6A (Zeta 1), isoform CRA_a** | **1.24890247** | **0.274530367** |
| **A0A024RDY3** | **Lysosomal-associated membrane protein 1, isoform CRA_a** | **1.173829644** | **0.66608928** |
| **A0A087WUL9** | **26S proteasome non-ATPase regulatory subunit 13** | **0.283471329** | **0.29826008** |
| **A0A087WVQ6** | **Clathrin heavy chain** | **0.997723363** | **0.996485388** |
| **A0A087WWM1** | **Mucin-1** | **1.524458648** | **0.13214307** |
| **A0A087WY68** | **Proprotein convertase subtilisin/kexin type 6** | **1.13637388** | **0.564543715** |
| **A0A096LPE2** | **SAA2-SAA4 readthrough** | **0.789258933** | **0.672819577** |
| **A0A0A0MSS8** | **Aldo-keto reductase family 1 member C3** | **1.497175995** | **0.239131608** |
| **A0A0A1I7H5** | **MHC class II antigen** | **0.419912512** | **0.113973625** |
| **A0A0A6YYG9** | **Protein ARPC4-TTLL3** | **1.756623196** | **0.271193486** |
| **A0A0A6YYL6** | **60S ribosomal protein L17** | **1.43120251** | **0.442196696** |
| **A0A0B4J2C3** | **Translationally-controlled tumor protein** | **1.013564816** | **0.982485208** |
| **A0A0C2T2M7** | **Uncharacterized protein** | **1.127705896** | **0.848171959** |
| **A0A0C4DG17** | **40S ribosomal protein SA** | **1.377418333** | **0.40146176** |
| **A0A0C4DGQ5** | **Calcium-activated neutral proteinase small subunit** | **0.650419822** | **0.502186836** |
| **A0A0C4DH26** | **Probable non-functional immunoglobulin kappa variable 6D-41** | **0.888975677** | **0.403111209** |
| **A0A0C9WPF5** | **Unplaced genomic scaffold K443scaffold_349, whole genome shotgun sequence** | **1.731539535** | **0.036606395** |
| **A0A0C9X1M1** | **Unplaced genomic scaffold K443scaffold_229, whole genome shotgun sequence** | **1.271462004** | **0.49401687** |
| **A0A0D2NHK4** | **Uncharacterized protein** | **1.178462013** | **0.561079904** |
| **A0A0D9SF53** | **RNA helicase** | **1.501389174** | **0.444380144** |
| **A0A0G2JH50** | **HLA class I histocompatibility antigen, C alpha chain** | **0.854015904** | **0.701058767** |
| **A0A0G2JRI9** | **Mucin-20** | **0.931746127** | **0.845248468** |
| **A0A0G2JS65** | **Mucin-4** | **0.721308837** | **0.52715634** |
| **A0A0J9YVR3** | **Alpha-S1-casein (Fragment)** | **1.244857175** | **0.038466852** |
| **A0A0K0K1J1** | **Epididymis secretory protein Li 2** | **0.954526384** | **0.588447319** |
| **A0A0S2Z3G9** | **Actinin alpha 4 isoform 1 (Fragment)** | **1.018450968** | **0.958247275** |
| **A0A0S2Z4G4** | **Tropomyosin 3 isoform 1 (Fragment)** | **1.012799027** | **0.969406523** |
| **A0A0U4BW16** | **Non-muscle myosin heavy chain 9** | **0.927194865** | **0.888849733** |
| **A0A0U4C4L3** | **Major histocompatibility complex class_II DQ alpha 1** | **0.68334642** | **0.436852801** |
| **A0A0U5EM55** | **Tumor necrosis factor ligand superfamily member** | **1.818595373** | **0.40992002** |
| **A0A0W0GCD8** | **Phospholipid-transporting ATPase** | **1.367273031** | **0.334319899** |
| **A0A140T9S8** | **HLA class II histocompatibility antigen, DQ beta 1 chain** | **0.493404544** | **0.278135111** |
| **A0A140VJC8** | **Amyloid-beta A4 protein** | **0.752581364** | **0.596432078** |
| **A0A140VJJ2** | **S-formylglutathione hydrolase** | **1.141602482** | **0.725586549** |
| **A0A140VJT8** | **Ribonuclease inhibitor** | **1.302035033** | **0.688698663** |
| **A0A140VJZ4** | **Ubiquitin carboxyl-terminal hydrolase** | **0.630766073** | **0.298312425** |
| **A0A140VK00** | **Testicular tissue protein Li 227** | **1.036122231** | **0.905625199** |
| **A0A140VK07** | **Testicular secretory protein Li 7** | **1.023285096** | **0.885997487** |
| **A0A140VK09** | **Testicular secretory protein Li 9** | **0.648736001** | **0.096256283** |
| **A0A140VK27** | **Leukotriene A(4) hydrolase** | **1.330817866** | **0.613019288** |
| **A0A140VK41** | **Ribose-phosphate diphosphokinase** | **1.083144156** | **0.725740122** |
| **A0A140VK83** | **Protein phosphatase 1, regulatory subunit 7, isoform CRA_b** | **5.560702959** | **0.261523155** |
| **A0A140VKC8** | **GDP-4-keto-6-deoxy-D-mannose-3,5-epimerase-4-reductase** | **0.998022498** | **0.981649742** |
| **A0A140VKH7** | **Testis tissue sperm-binding protein Li 94mP** | **2.264520194** | **0.377992352** |
| **A0A146HHY6** | **Zn(2)-C6 fungal-type domain-containing protein** | **1.497311627** | **0.448824859** |
| **A0A1B0GVI3** | **Keratin, type I cytoskeletal 10** | **0.672979509** | **0.064025026** |
| **A0A1B0GW77** | **Aldehyde dehydrogenase (NAD(+))** | **2.871714837** | **0.034486162** |
| **A0A1B1CYC5** | **Vitamin D binding protein (Fragment)** | **0.800744074** | **0.53743584** |
| **A0A1C7CYX9** | **Dihydropyrimidinase-related protein 2** | **0.72228795** | **0.411301441** |
| **A0A1L2BU40** | **Anti-staphylococcal enterotoxin E variable region lambda chain (Fragment)** | **0.825982014** | **0.4480063** |
| **A0A1W2PNV4** | **Uncharacterized protein** | **1.313576161** | **0.542057817** |
| **A0A1W2PRS1** | **Lysosome membrane protein 2 (Fragment)** | **4.339686489** | **0.012024746** |
| **A0A284QLK7** | **Uncharacterized protein** | **0.668714651** | **0.313467416** |
| **A0A2R8Y5V9** | **Tropomyosin alpha-4 chain** | **0.742255358** | **0.41525965** |
| **A0A2R8Y5Y7** | **60S ribosomal protein L9** | **1.469797913** | **0.349383681** |
| **A0A2R8Y6G6** | **2-phospho-D-glycerate hydro-lyase** | **1.120787619** | **0.490740304** |
| **A0A2S0BDD1** | **Antithrombin-III** | **0.786117259** | **0.227896148** |
| **A0A2U3TZU2** | **Glucose-6-phosphate isomerase** | **0.774750369** | **0.035045557** |
| **A0A384MDQ7** | **Epididymis secretory sperm binding protein** | **0.839164583** | **0.350645973** |
| **A0A384MDV6** | **Carboxypeptidase Z, isoform CRA_b** | **0.646678065** | **0.184060889** |
| **A0A384MEF1** | **Actin-depolymerizing factor** | **0.713852843** | **0.193490048** |
| **A0A384MQW4** | **Epididymis secretory sperm binding protein** | **0.588441627** | **0.433547508** |
| **A0A384MTQ5** | **Terpene cyclase/mutase family member** | **1.237995083** | **0.748908995** |
| **A0A384N6G7** | **GlcNAc kinase** | **1.478239474** | **0.313221973** |
| **A0A384NKS6** | **Clusterin** | **0.610445386** | **0.070959468** |
| **A0A384NL58** | **Heterogeneous nuclear ribonucleoproteins A2/B1** | **0.582233457** | **0.225948639** |
| **A0A384NY14** | **Dicarbonyl/L-xylulose reductase, isoform CRA_b** | **6.642281149** | **0.197589299** |
| **A0A3B0J271** | **Adiponectin P** | **0.511847951** | **0.164144507** |
| **A0A3B3IRV5** | **Heme-binding protein 1 (Fragment)** | **0.971674721** | **0.953584456** |
| **A0A3B3ISQ4** | **Epidermal growth factor receptor kinase substrate 8-like protein 2** | **0.944627949** | **0.677723991** |
| **A0A3B3ITK7** | **Phosphoglucomutase-1** | **0.71318254** | **0.570090545** |
| **A0A409VAT6** | **Uncharacterized protein** | **0.516849968** | **0.320679886** |
| **A0A409XFV3** | **Uncharacterized protein** | **1.792491054** | **0.308815453** |
| **A0A409YAF2** | **RRM domain-containing protein** | **0.948275525** | **0.750116409** |
| **A0A409YQI8** | **Uncharacterized protein** | **3.39369479** | **0.1630015** |
| **A0A494C039** | **Hypoxia up-regulated protein 1** | **1.296025593** | **0.395749279** |
| **A0A494C0F3** | **EXPERA domain-containing protein** | **0.533944521** | **0.412636688** |
| **A0A499FI48** | **Protein disulfide-isomerase** | **0.747734767** | **0.540672015** |
| **A0A4Q2DK56** | **RRM domain-containing protein** | **0.628671909** | **0.450931069** |
| **A0A4Q2DSU6** | **Uncharacterized protein** | **3.846666706** | **0.162839659** |
| **A0A550BYR1** | **Uncharacterized protein** | **0.686009985** | **0.43844368** |
| **A0A590UJ43** | **Casein kinase I isoform alpha** | **1.035372698** | **0.934021463** |
| **A0A5C2FU65** | **IGL c528_light_IGKV2D-28_IGKJ2 (Fragment)** | **2.111890627** | **0.310397983** |
| **A0A5C2FW95** | **IGL c1358_light_IGKV1D-39_IGKJ2 (Fragment)** | **1.39463334** | **0.391660699** |
| **A0A5C2G2M1** | **IGL c3521_light_IGKV3-15_IGKJ2 (Fragment)** | **0.547282064** | **0.062997832** |
| **A0A5C2G585** | **IGL c1483_light_IGKV1-5_IGKJ2 (Fragment)** | **0.508054926** | **0.033971462** |
| **A0A5C2GFN9** | **IGH + IGL c179_light_IGKV3-11_IGKJ2 (Fragment)** | **0.782902284** | **0.366651357** |
| **A0A5C2GIN5** | **IG c528_heavy_IGHV3-15_IGHD2-8_IGHJ4 (Fragment)** | **0.710275185** | **0.153117343** |
| **A0A5C2GJR5** | **IG c837_light_IGKV1-39_IGKJ2 (Fragment)** | **0.565674384** | **0.022648501** |
| **A0A5C2GJY2** | **IG c267_light_IGKV3-20_IGKJ1 (Fragment)** | **0.681657006** | **0.282351398** |
| **A0A5C2GLT5** | **IG c1145_heavy_IGHV1-18_IGHD3-22_IGHJ6 (Fragment)** | **0.471333727** | **0.022930468** |
| **A0A5C2GMT3** | **IG c1332_heavy_IGHV3-21_IGHD2-2_IGHJ6 (Fragment)** | **0.346998382** | **0.058092525** |
| **A0A5C2GS56** | **IG c325_light_IGLV1-44_IGLJ2 (Fragment)** | **3.007613329** | **0.120687774** |
| **A0A5C2GSH3** | **IG c1440_heavy_IGHV3-30_IGHD5-12_IGHJ4 (Fragment)** | **0.441304393** | **0.05692836** |
| **A0A5C2GUW3** | **IG c1221_light_IGKV1-27_IGKJ1 (Fragment)** | **0.754488952** | **0.020009775** |
| **A0A5C3KIB7** | **ATP-binding cassette transporter** | **0.769312159** | **0.734031689** |
| **A0A5F9ZHM4** | **L-lactate dehydrogenase** | **1.51886984** | **0.529580753** |
| **A0A5K1VW95** | **Malate dehydrogenase** | **2.06446661** | **0.158380333** |
| **A0A679KL62** | **ACX82 (Fragment)** | **0.578215387** | **0.063483099** |
| **A0A6A4HJB2** | **Uncharacterized protein (Fragment)** | **0.897838384** | **0.71012859** |
| **A0A6Q8PGK1** | **Heat shock 27 kDa protein** | **1.141335875** | **0.252066387** |
| **A0A6Q8PH84** | **Ras-related protein Rab-7a** | **1.108398348** | **0.883074777** |
| **A0A7I2V3S3** | **Eukaryotic translation initiation factor 3 subunit E** | **0.640656496** | **0.469148374** |
| **A0A7I2V3V7** | **Synaptobrevin homolog YKT6** | **1.331247769** | **0.295175769** |
| **A0A7P0S5H5** | **40S ribosomal protein S20 (Fragment)** | **1.330543247** | **0.457540836** |
| **A0A7P0T9C4** | **Cytoplasmic dynein 1 heavy chain 1** | **1.491446613** | **0.407561322** |
| **A0A7P0TA71** | **Protein disulfide-isomerase** | **0.855130771** | **0.713150417** |
| **A0A7P0TAE9** | **Calnexin** | **1.895735275** | **0.420461991** |
| **A0A7P0TAT8** | **Endoplasmin** | **0.114605154** | **0.125467821** |
| **A0A7S5C3A4** | **IGH c2314_heavy_IGHV3-7_IGHD3-16_IGHJ4 (Fragment)** | **0.925032413** | **0.7234942** |
| **A0A7S5C4U5** | **IGH c4086_heavy_IGHV3-53_IGHD3-9_IGHJ6 (Fragment)** | **5.248457304** | **0.157847237** |
| **A0A7S5EXD1** | **IGH c1503_heavy_IGHV3-49_IGHD1-1_IGHJ6 (Fragment)** | **0.450833516** | **0.016915508** |
| **A1A4E9** | **Keratin 13** | **0.15130342** | **0.423992582** |
| **A2A3R6** | **40S ribosomal protein S6** | **1.56329499** | **0.285841638** |
| **A2J1N5** | **Rheumatoid factor RF-ET6 (Fragment)** | **0.346707139** | **0.025399727** |
| **A4D125** | **Sclerostin domain containing 1** | **0.799994669** | **0.422836562** |
| **A4D1B1** | **Glycoprotein IIIb** | **2.09115145** | **0.621183864** |
| **A4D2P0** | **Ras-related C3 botulinum toxin substrate 1 (Rho family, small GTP binding protein Rac1)** | **0.852740897** | **0.734146538** |
| **A4QPH0** | **SLC5A1 protein (Fragment)** | **4.522857905** | **0.302948109** |
| **A5PL27** | **CP protein** | **1.107323684** | **0.519589024** |
| **A5PLK9** | **Metalloendopeptidase** | **0.818234609** | **0.161972463** |
| **A6NMH8** | **Tetraspanin** | **1.024403611** | **0.953115156** |
| **A6XGL1** | **Transthyretin** | **1.118071306** | **0.486203284** |
| **A7BI36** | **p180/ribosome receptor** | **0.560607529** | **0.062993462** |
| **A8K088** | **RNA helicase** | **1.110806427** | **0.734684508** |
| **A8K0T9** | **F-actin-capping protein subunit alpha** | **0.812610883** | **0.681087506** |
| **A8K287** | **Synaptosomal-associated protein** | **0.914194616** | **0.73987249** |
| **A8K2M5** | **Lipoprotein lipase** | **0.916144421** | **0.680402153** |
| **A8K2Q6** | **Peptidyl-prolyl cis-trans isomerase** | **1.378668243** | **0.114200457** |
| **A8K3C3** | **T-complex protein 1 subunit delta** | **1.257906033** | **0.690715876** |
| **A8K3K1** | **cDNA FLJ78096, highly similar to Homo sapiens actin, alpha, cardiac muscle (ACTC), mRNA** | **1.291670483** | **0.355019334** |
| **A8K486** | **Peptidyl-prolyl cis-trans isomerase** | **1.220425384** | **0.465211172** |
| **A8K491** | **cDNA FLJ76037, highly similar to Homo sapiens matrilin 3 (MATN3), mRNA** | **0.869778223** | **0.499057273** |
| **A8K4Z4** | **60S acidic ribosomal protein P0** | **1.118818217** | **0.74827522** |
| **A8K669** | **Asparaginyl endopeptidase** | **0.288391317** | **0.039518969** |
| **A8K690** | **cDNA FLJ76863, highly similar to Homo sapiens stress-induced-phosphoprotein 1 (Hsp70/Hsp90-organizing protein) (STIP1), mRNA** | **2.441516357** | **0.016105823** |
| **A8K7Q1** | **Nucleobindin-1** | **0.390836302** | **0.000159388** |
| **A8K9C4** | **Elongation factor 1-alpha** | **1.164350997** | **0.746390134** |
| **A8K9T3** | **cDNA FLJ76467, highly similar to Homo sapiens acyl-CoA synthetase long-chain family member 1 (ACSL1), mRNA** | **0.341092299** | **0.219161381** |
| **A8MUS3** | **60S ribosomal protein L23a** | **1.773636141** | **0.688652159** |
| **B0YIW2** | **Apolipoprotein C-III** | **0.432570437** | **0.521592138** |
| **B2R491** | **40S ribosomal protein S4** | **1.158192239** | **0.810391941** |
| **B2R4C0** | **60S ribosomal protein L18a** | **2.573738003** | **0.318400183** |
| **B2R4M6** | **Protein S100** | **1.052691045** | **0.857645245** |
| **B2R4R0** | **Histone H4** | **1.156659345** | **0.574112986** |
| **B2R5M8** | **Isocitrate dehydrogenase [NADP]** | **0.92449203** | **0.81747417** |
| **B2R5T2** | **cDNA, FLJ92608, highly similar to Homo sapiens aldehyde dehydrogenase 1 family, member A3 (ALDH1A3), mRNA** | **0.756619358** | **0.645165987** |
| **B2R657** | **Annexin** | **0.651999407** | **0.470664324** |
| **B2R6A3** | **Na(+)/H(+) exchange regulatory cofactor NHE-RF** | **0.828908138** | **0.658708684** |
| **B2R6D0** | **26S proteasome non-ATPase regulatory subunit 1** | **0.597646344** | **0.457848228** |
| **B2R6J2** | **cDNA, FLJ92973, highly similar to Homo sapiens villin 2 (ezrin) (VIL2), mRNA** | **1.117378039** | **0.723037805** |
| **B2R761** | **Acetyl-CoA C-myristoyltransferase** | **0.939525313** | **0.899556696** |
| **B2R774** | **cDNA, FLJ93313, highly similar to Homo sapiens lectin, mannose-binding, 1 (LMAN1), mRNA** | **0.868885643** | **0.769110216** |
| **B2R7F8** | **Plasminogen** | **0.626325078** | **0.021449484** |
| **B2R888** | **Monocyte differentiation antigen CD14** | **1.103608012** | **0.749942445** |
| **B2R8J1** | **Transporter** | **0.733446771** | **0.3386444** |
| **B2R9U2** | **Peptidylprolyl isomerase** | **0.856330605** | **0.680865575** |
| **B2R9X3** | **GDP-D-mannose dehydratase** | **1.09647678** | **0.82373449** |
| **B2RBE0** | **cDNA, FLJ95462, highly similar to Homo sapiens fatty-acid-Coenzyme A ligase, long-chain 3 (FACL3),mRNA** | **0.962289515** | **0.83517033** |
| **B2RBR9** | **cDNA, FLJ95650, highly similar to Homo sapiens karyopherin (importin) beta 1 (KPNB1), mRNA** | **1.366391438** | **0.381657898** |
| **B2RBS8** | **cDNA, FLJ95666, highly similar to Homo sapiens albumin (ALB), mRNA** | **0.820140312** | **0.242739382** |
| **B2RDW1** | **40S ribosomal protein S27a** | **0.927983117** | **0.800822102** |
| **B3KQF4** | **Metalloproteinase inhibitor 1** | **1.157439906** | **0.662556371** |
| **B3KQF5** | **Calumenin** | **1.022623978** | **0.962384459** |
| **B3KS79** | **cDNA FLJ35730 fis, clone TESTI2003131, highly similar to ALPHA-1-ANTICHYMOTRYPSIN** | **0.390437983** | **0.037523293** |
| **B3KSH1** | **Eukaryotic translation initiation factor 3 subunit F** | **0.978290506** | **0.948400423** |
| **B3VMW0** | **Lactotransferrin** | **0.707586726** | **0.180134535** |
| **B4DE59** | **Junction plakoglobin** | **0.604043943** | **0.423986248** |
| **B4DJ30** | **cDNA FLJ61290, highly similar to Neutral alpha-glucosidase AB** | **1.206082833** | **0.466654133** |
| **B4DJQ5** | **Glucosidase 2 subunit beta** | **1.142385096** | **0.643061746** |
| **B4DLV7** | **Rab GDP dissociation inhibitor** | **0.816893751** | **0.384327616** |
| **B4DPD5** | **Ubiquitin thioesterase** | **0.873171496** | **0.859970076** |
| **B4DUH8** | **Carbonic anhydrase** | **0.746674199** | **0.060970483** |
| **B4DUP2** | **UTP--glucose-1-phosphate uridylyltransferase** | **1.653588582** | **0.385384963** |
| **B4DW34** | **Acid sphingomyelinase-like phosphodiesterase** | **0.716776313** | **0.389861746** |
| **B4DWA6** | **F-actin-capping protein subunit beta** | **0.838864229** | **0.907479343** |
| **B4DX14** | **cDNA FLJ53231, highly similar to 4-trimethylaminobutyraldehyde dehydrogenase** | **38.59328661** | **0.12831094** |
| **B4DX19** | **Paraoxonase** | **1.254100686** | **0.667095623** |
| **B4DY90** | **Tubulin beta chain** | **1.165963125** | **0.331899792** |
| **B4E0X1** | **Beta-2-microglobulin** | **0.480258242** | **0.420322009** |
| **B4E0Z6** | **cDNA FLJ59809, highly similar to Bone marrow stromal antigen 2** | **0.218489033** | **0.281431643** |
| **B4E1B2** | **Beta-1 metal-binding globulin** | **0.679071262** | **0.120574943** |
| **B4E1C2** | **Bradykinin** | **4.603105474** | **0.338434556** |
| **B4E1U9** | **Cell division control protein 42 homolog** | **0.604367824** | **0.138537875** |
| **B4E1Z4** | **C3/C5 convertase** | **0.613900782** | **0.349365812** |
| **B5ME19** | **Eukaryotic translation initiation factor 3 subunit C-like protein** | **1.826492423** | **0.303291682** |
| **B7Z1F8** | **cDNA FLJ53025, highly similar to Complement C4-B** | **0.886768339** | **0.531221953** |
| **B7Z1K5** | **Tubulin alpha chain** | **1.021368154** | **0.929573707** |
| **B7Z351** | **Secreted phosphoprotein 1 variant 6** | **0.976318207** | **0.771793046** |
| **B7Z6Z4** | **Myosin light polypeptide 6** | **1.264126292** | **0.199053304** |
| **B7Z831** | **cDNA FLJ55176, highly similar to G-protein coupled receptor family C group 5 member B** | **0.892379159** | **0.464903211** |
| **B7Z8E7** | **cDNA FLJ55400, highly similar to Protein** | **4.291992268** | **0.386366218** |
| **B7Z8Q2** | **Alpha-2-HS-glycoprotein** | **0.46043362** | **0.097411417** |
| **B7ZKQ8** | **Podocalyxin** | **0.588691012** | **0.077552942** |
| **B7ZMD7** | **Alpha-amylase** | **0.826473308** | **0.475190235** |
| **B8ZWD9** | **Diazepam binding inhibitor, splice form 1D(2)** | **1.144522602** | **0.515622446** |
| **B9EJA8** | **Mannose receptor, C type 1-like 1** | **0.947477452** | **0.821276762** |
| **C0JYY2** | **Apolipoprotein B (Including Ag(X) antigen)** | **1.106702146** | **0.483088785** |
| **C9J660** | **Inactive C-alpha-formylglycine-generating enzyme 2** | **1.383952193** | **0.285278836** |
| **C9JC84** | **Fibrinogen gamma chain** | **0.637987779** | **0.036707219** |
| **C9JF17** | **Apolipoprotein D (Fragment)** | **0.719253647** | **0.290373419** |
| **D0PNI1** | **Epididymis luminal protein 4** | **0.439723442** | **0.352907971** |
| **D3DQ70** | **SERPINE1 mRNA binding protein 1, isoform CRA_d** | **1.640523239** | **0.322365166** |
| **D3DRP5** | **Chromosome 9 open reading frame 19, isoform CRA_a (Fragment)** | **0.77185765** | **0.350377714** |
| **D3DRR6** | **Inter-alpha (Globulin) inhibitor H2, isoform CRA_a** | **0.916639778** | **0.517527091** |
| **D6RF35** | **Gc-globulin** | **0.756369203** | **0.206521018** |
| **D6RH31** | **Nephronectin (Fragment)** | **0.942556568** | **0.769902923** |
| **D8PR62** | **Uncharacterized protein (Fragment)** | **0.895477079** | **0.343054567** |
| **D9IAI1** | **Epididymis secretory protein Li 34** | **1.106834315** | **0.369454078** |
| **D9ZGG2** | **Vitronectin** | **0.874401173** | **0.539394177** |
| **E9PAV3** | **Nascent polypeptide-associated complex subunit alpha, muscle-specific form** | **1.451798274** | **0.238810703** |
| **E9PDQ1** | **Alpha-S1-casein** | **1.288741975** | **0.105278347** |
| **E9PGN7** | **Plasma protease C1 inhibitor** | **1.077365237** | **0.536502509** |
| **E9PHZ5** | **Cathepsin B** | **0.566581056** | **0.287178496** |
| **E9PK25** | **Cofilin, non-muscle isoform** | **0.926137711** | **0.41084811** |
| **E9PRY8** | **Elongation factor 1-delta** | **1.534298928** | **0.343854257** |
| **F5GXT1** | **Tetraspanin** | **1.326073753** | **0.156340976** |
| **F8W727** | **60S ribosomal protein L32** | **1.072638894** | **0.84455768** |
| **G3V5Z7** | **Proteasome subunit alpha type** | **2.023020622** | **0.311382503** |
| **G8H6I3** | **Endocrine and exocrine protein** | **0.944724401** | **0.816001671** |
| **G9K388** | **YWHAE/FAM22A fusion protein (Fragment)** | **0.460079706** | **0.066292645** |
| **H0Y449** | **Y-box-binding protein 1 (Fragment)** | **1.681145469** | **0.221376684** |
| **H0Y858** | **Toll-like receptor 9 (Fragment)** | **0.980541016** | **0.949228721** |
| **H0Y8K1** | **Alpha-S1-casein (Fragment)** | **0.82491826** | **0.576568412** |
| **H0YCU0** | **Chordin-like protein 2 (Fragment)** | **0.473171146** | **0.215524605** |
| **H0YI09** | **Methyltransferase-like protein 7A (Fragment)** | **0.525165993** | **0.072261937** |
| **H0YMW4** | **Annexin** | **1.821137404** | **0.384583631** |
| **H3BN98** | **40S ribosomal protein S15a (Fragment)** | **0.902986461** | **0.470058484** |
| **H3BNC9** | **40S ribosomal protein S17** | **0.734898389** | **0.365602649** |
| **H3BTH8** | **Hyaluronan and proteoglycan link protein 3** | **1.02003845** | **0.960910828** |
| **H6VRG1** | **Cytokeratin-1** | **0.908434119** | **0.43731076** |
| **H7BZJ3** | **Protein disulfide-isomerase A3 (Fragment)** | **0.821180072** | **0.605510903** |
| **I4AY87** | **Epididymis secretory sperm binding protein (Fragment)** | **0.830271079** | **0.250145671** |
| **I7JB59** | **ATP-binding cassette sub-family G member 2** | **0.969886853** | **0.924465058** |
| **J3KPS3** | **Fructose-bisphosphate aldolase** | **1.489687356** | **0.401476026** |
| **J3KQE5** | **GTP-binding nuclear protein Ran (Fragment)** | **0.67838573** | **0.005455565** |
| **J3QQ67** | **60S ribosomal protein L18 (Fragment)** | **0.448437392** | **0.414691212** |
| **J3QQX2** | **Rho GDP-dissociation inhibitor 1** | **2.004956449** | **0.015161223** |
| **J3QSU6** | **Tenascin** | **0.571664588** | **0.303843414** |
| **J9R021** | **Eukaryotic translation initiation factor 3 subunit A** | **1.010584798** | **0.975498207** |
| **K5XX64** | **Non-specific serine/threonine protein kinase** | **0.699959893** | **0.412669445** |
| **K7EJ20** | **Glutathione peroxidase** | **1.559017878** | **0.315389875** |
| **K7ELC2** | **40S ribosomal protein S15** | **1.115752715** | **0.756263008** |
| **K7ELC7** | **60S ribosomal protein L27 (Fragment)** | **1.460419323** | **0.269826861** |
| **K7ER00** | **Phenylalanine--tRNA ligase** | **0.681044559** | **0.649138102** |
| **K9JA46** | **Epididymis luminal secretory protein 52** | **1.530824572** | **0.165775597** |
| **M0QYS1** | **60S ribosomal protein L13a (Fragment)** | **0.871297504** | **0.76032425** |
| **M1V485** | **Tyrosine-protein kinase receptor** | **0.524308466** | **0.20610793** |
| **M1VKI3** | **Tyrosine-protein kinase receptor** | **0.679050769** | **0.451752077** |
| **M1VPF6** | **Tyrosine-protein kinase receptor** | **0.673250736** | **0.429094927** |
| **O00231** | **26S proteasome non-ATPase regulatory subunit 11** | **0.779825493** | **0.651860087** |
| **O00299** | **Chloride intracellular channel protein 1** | **1.105435061** | **0.778274258** |
| **O00300** | **Tumor necrosis factor receptor superfamily member 11B** | **0.542981057** | **0.149689677** |
| **O00391** | **Sulfhydryl oxidase 1** | **0.670421765** | **0.411950653** |
| **O00468** | **Agrin** | **0.435278513** | **0.048526396** |
| **O15144** | **Actin-related protein 2/3 complex subunit 2** | **0.861638067** | **0.7630894** |
| **O15371** | **Eukaryotic translation initiation factor 3 subunit D** | **0.851328434** | **0.723097426** |
| **O43175** | **D-3-phosphoglycerate dehydrogenase** | **0.594221055** | **0.261926627** |
| **O43242** | **26S proteasome non-ATPase regulatory subunit 3** | **0.345703025** | **0.034891007** |
| **O43488** | **Aflatoxin B1 aldehyde reductase member 2** | **0.933999267** | **0.680204317** |
| **O60664** | **Perilipin-3** | **1.261003068** | **0.462132871** |
| **O60763** | **General vesicular transport factor p115** | **1.772132302** | **0.407026267** |
| **O75477** | **Erlin-1** | **0.605116928** | **0.424829784** |
| **P00167** | **Cytochrome b5** | **1.017788706** | **0.960355763** |
| **P00709** | **Alpha-lactalbumin** | **0.812629685** | **0.021824223** |
| **P00734** | **Prothrombin** | **0.635333272** | **0.076055332** |
| **P00738** | **Haptoglobin** | **0.821222613** | **0.556049752** |
| **P01023** | **Alpha-2-macroglobulin** | **0.333425415** | **0.030028517** |
| **P01024** | **Complement C3** | **0.497845806** | **0.043429833** |
| **P01591** | **Immunoglobulin J chain** | **0.550935154** | **0.007247541** |
| **P01833** | **Polymeric immunoglobulin receptor** | **0.610870447** | **0.031114304** |
| **P01871** | **Immunoglobulin heavy constant mu** | **0.747980397** | **0.009366829** |
| **P01877** | **Immunoglobulin heavy constant alpha 2** | **0.693082015** | **0.027232089** |
| **P02647** | **Apolipoprotein A-I** | **1.045732224** | **0.775322426** |
| **P02649** | **Apolipoprotein E** | **1.099774327** | **0.663877926** |
| **P02671** | **Fibrinogen alpha chain** | **0.540750423** | **0.012334968** |
| **P02675** | **Fibrinogen beta chain** | **1.735821896** | **0.287774994** |
| **P02750** | **Leucine-rich alpha-2-glycoprotein** | **0.641078421** | **0.331326575** |
| **P02751** | **Fibronectin** | **1.448195743** | **0.586481734** |
| **P02760** | **Protein AMBP** | **1.612941425** | **0.550625916** |
| **P02787** | **Serotransferrin** | **0.185949642** | **0.004537587** |
| **P02790** | **Hemopexin** | **0.812925043** | **0.500937799** |
| **P04003** | **C4b-binding protein alpha chain** | **0.497807025** | **0.204921978** |
| **P04040** | **Catalase** | **0.897147651** | **0.802900437** |
| **P04083** | **Annexin A1** | **0.538551517** | **0.257258075** |
| **P04179** | **Superoxide dismutase [Mn], mitochondrial** | **0.770494298** | **0.64968786** |
| **P04406** | **Glyceraldehyde-3-phosphate dehydrogenase** | **0.960316054** | **0.881822862** |
| **P04843** | **Dolichyl-diphosphooligosaccharide--protein glycosyltransferase subunit 1** | **0.487722918** | **0.225638131** |
| **P05109** | **Protein S100-A8** | **1.198050964** | **0.831412568** |
| **P05387** | **60S acidic ribosomal protein P2** | **1.131568392** | **0.818055345** |
| **P05413** | **Fatty acid-binding protein, heart** | **0.805072262** | **0.536029075** |
| **P05546** | **Heparin cofactor 2** | **0.38965312** | **0.067134787** |
| **P05787** | **Keratin, type II cytoskeletal 8** | **1.130851352** | **0.499457767** |
| **P06727** | **Apolipoprotein A-IV** | **0.922445168** | **0.614123605** |
| **P07498** | **Kappa-casein** | **1.105068518** | **0.370100358** |
| **P07814** | **Bifunctional glutamate/proline--tRNA ligase** | **0.842170441** | **0.25387872** |
| **P08729** | **Keratin, type II cytoskeletal 7** | **0.697901439** | **0.334973908** |
| **P08758** | **Annexin A5** | **0.507844793** | **0.24705551** |
| **P08962** | **CD63 antigen** | **0.425140512** | **0.014870002** |
| **P09543** | **2',3'-cyclic-nucleotide 3'-phosphodiesterase** | **0.12084245** | **0.313248056** |
| **P0C0L4** | **Complement C4-A** | **0.744222284** | **0.119361319** |
| **P0C0L5** | **Complement C4-B** | **0.899281689** | **0.759966347** |
| **P0DOX2** | **Immunoglobulin alpha-2 heavy chain** | **0.36633655** | **0.038221715** |
| **P10586** | **Receptor-type tyrosine-protein phosphatase F** | **0.413926042** | **0.116912916** |
| **P10599** | **Thioredoxin** | **1.009635578** | **0.965145234** |
| **P11234** | **Ras-related protein Ral-B** | **0.7173638** | **0.612022343** |
| **P12273** | **Prolactin-inducible protein** | **0.435674964** | **0.487357303** |
| **P13639** | **Elongation factor 2** | **1.442410494** | **0.055052235** |
| **P13987** | **CD59 glycoprotein** | **0.479410342** | **0.013143307** |
| **P14868** | **Aspartate--tRNA ligase, cytoplasmic** | **1.972243276** | **0.092605884** |
| **P15291** | **Beta-1,4-galactosyltransferase 1** | **1.059853005** | **0.462818862** |
| **P15531** | **Nucleoside diphosphate kinase A** | **0.891447862** | **0.796585453** |
| **P15880** | **40S ribosomal protein S2** | **1.900255522** | **0.087849327** |
| **P17858** | **ATP-dependent 6-phosphofructokinase, liver type** | **0.932720271** | **0.866298016** |
| **P17987** | **T-complex protein 1 subunit alpha** | **0.55352146** | **0.089739969** |
| **P18065** | **Insulin-like growth factor-binding protein 2** | **1.982250648** | **0.552496782** |
| **P18085** | **ADP-ribosylation factor 4** | **0.903966402** | **0.69308196** |
| **P19652** | **Alpha-1-acid glycoprotein 2** | **1.170718515** | **0.602524734** |
| **P19827** | **Inter-alpha-trypsin inhibitor heavy chain H1** | **0.773018261** | **0.449416919** |
| **P19835** | **Bile salt-activated lipase** | **1.131903881** | **0.324542358** |
| **P20061** | **Transcobalamin-1** | **0.98356215** | **0.961769285** |
| **P22079** | **Lactoperoxidase** | **1.459589906** | **0.156925612** |
| **P22352** | **Glutathione peroxidase 3** | **0.893072865** | **0.596383837** |
| **P23284** | **Peptidyl-prolyl cis-trans isomerase B** | **1.027090494** | **0.928630358** |
| **P23396** | **40S ribosomal protein S3** | **0.433937326** | **0.508793119** |
| **P23526** | **Adenosylhomocysteinase** | **1.153300198** | **0.597672456** |
| **P25705** | **ATP synthase subunit alpha, mitochondrial** | **0.953859864** | **0.944567805** |
| **P25774** | **Cathepsin S** | **0.568234552** | **0.048095553** |
| **P25788** | **Proteasome subunit alpha type-3** | **0.979402531** | **0.938630492** |
| **P26038** | **Moesin** | **0.858322381** | **0.594557983** |
| **P26373** | **60S ribosomal protein L13** | **1.960699285** | **0.514727369** |
| **P26639** | **Threonine--tRNA ligase 1, cytoplasmic** | **1.31143798** | **0.120049231** |
| **P26641** | **Elongation factor 1-gamma** | **1.175969876** | **0.639643268** |
| **P27348** | **14-3-3 protein theta** | **1.003162485** | **0.99234173** |
| **P28676** | **Grancalcin** | **1.847602527** | **0.184524096** |
| **P29401** | **Transketolase** | **1.999368797** | **0.4922668** |
| **P29966** | **Myristoylated alanine-rich C-kinase substrate** | **0.84960809** | **0.569669757** |
| **P30041** | **Peroxiredoxin-6** | **0.790068692** | **0.633701898** |
| **P31946** | **14-3-3 protein beta/alpha** | **1.19936005** | **0.309619935** |
| **P32119** | **Peroxiredoxin-2** | **0.415966159** | **0.182157677** |
| **P33908** | **Mannosyl-oligosaccharide 1,2-alpha-mannosidase IA** | **2.792878175** | **0.035985125** |
| **P35030** | **Trypsin-3** | **1.594474774** | **0.24887266** |
| **P35268** | **60S ribosomal protein L22** | **1.317650157** | **0.63400751** |
| **P35527** | **Keratin, type I cytoskeletal 9** | **0.410003805** | **0.20971262** |
| **P35908** | **Keratin, type II cytoskeletal 2 epidermal** | **0.243190443** | **0.234072172** |
| **P46776** | **60S ribosomal protein L27a** | **0.714538336** | **0.197695855** |
| **P46777** | **60S ribosomal protein L5** | **1.345247596** | **0.228134813** |
| **P47710** | **Alpha-S1-casein** | **0.973843597** | **0.901235894** |
| **P47989** | **Xanthine dehydrogenase/oxidase** | **0.948199179** | **0.802735722** |
| **P48643** | **T-complex protein 1 subunit epsilon** | **2.6712386** | **0.150944176** |
| **P49006** | **MARCKS-related protein** | **0.503085071** | **0.085743207** |
| **P49327** | **Fatty acid synthase** | **1.622145794** | **0.177320922** |
| **P49788** | **Retinoic acid receptor responder protein 1** | **1.189089892** | **0.36303933** |
| **P49908** | **Selenoprotein P** | **0.876145056** | **0.443038116** |
| **P50502** | **Hsc70-interacting protein** | **1.197828312** | **0.519089826** |
| **P50990** | **T-complex protein 1 subunit theta** | **1.030011627** | **0.951290484** |
| **P51993** | **4-galactosyl-N-acetylglucosaminide 3-alpha-L-fucosyltransferase FUT6** | **0.994707918** | **0.979315001** |
| **P52209** | **6-phosphogluconate dehydrogenase, decarboxylating** | **0.833692827** | **0.657321553** |
| **P53634** | **Dipeptidyl peptidase 1** | **0.437033174** | **0.203397028** |
| **P54136** | **Arginine--tRNA ligase, cytoplasmic** | **0.937713986** | **0.911734194** |
| **P54920** | **Alpha-soluble NSF attachment protein** | **0.634180587** | **0.439078362** |
| **P55072** | **Transitional endoplasmic reticulum ATPase** | **1.844210045** | **0.429925352** |
| **P57721** | **Poly(rC)-binding protein 3** | **0.502315459** | **0.383596304** |
| **P59665** | **Neutrophil defensin 1** | **0.606047452** | **0.260319461** |
| **P60709** | **Actin, cytoplasmic 1** | **1.0940869** | **0.772282021** |
| **P61160** | **Actin-related protein 2** | **1.394188273** | **0.304805574** |
| **P61224** | **Ras-related protein Rap-1b** | **0.660136295** | **0.168025482** |
| **P61513** | **60S ribosomal protein L37a** | **0.542334363** | **0.154752925** |
| **P61626** | **Lysozyme C** | **0.877317896** | **0.428166703** |
| **P61764** | **Syntaxin-binding protein 1** | **0.561492918** | **0.160094496** |
| **P62249** | **40S ribosomal protein S16** | **0.941709463** | **0.730255303** |
| **P62263** | **40S ribosomal protein S14** | **1.454841985** | **0.09537701** |
| **P62269** | **40S ribosomal protein S18** | **1.198902238** | **0.566612648** |
| **P62277** | **40S ribosomal protein S13** | **1.057050834** | **0.6881125** |
| **P62280** | **40S ribosomal protein S11** | **0.753636375** | **0.640719569** |
| **P62424** | **60S ribosomal protein L7a** | **0.831067614** | **0.051566363** |
| **P62851** | **40S ribosomal protein S25** | **0.969104852** | **0.962075561** |
| **P62879** | **Guanine nucleotide-binding protein G(I)/G(S)/G(T) subunit beta-2** | **1.361417074** | **0.149174033** |
| **P62906** | **60S ribosomal protein L10a** | **1.340611447** | **0.294716397** |
| **P62913** | **60S ribosomal protein L11** | **0.160171079** | **0.40304663** |
| **P62917** | **60S ribosomal protein L8** | **1.315293115** | **0.462636402** |
| **P63244** | **Receptor of activated protein C kinase 1** | **1.264939837** | **0.084508697** |
| **P68371** | **Tubulin beta-4B chain** | **0.186220846** | **0.34669852** |
| **P80303** | **Nucleobindin-2** | **0.565948341** | **0.005410324** |
| **Q02809** | **Procollagen-lysine,2-oxoglutarate 5-dioxygenase 1** | **0.613467236** | **0.45535043** |
| **Q05D08** | **PA2G4 protein (Fragment)** | **0.445366232** | **0.030756542** |
| **Q06210** | **Glutamine--fructose-6-phosphate aminotransferase [isomerizing] 1** | **1.041824576** | **0.738528598** |
| **Q06481** | **Amyloid-like protein 2** | **0.918743195** | **0.859138812** |
| **Q06830** | **Peroxiredoxin-1** | **1.13918986** | **0.479760558** |
| **Q08380** | **Galectin-3-binding protein** | **0.884251841** | **0.474963765** |
| **Q08431** | **Lactadherin** | **0.923509574** | **0.322495042** |
| **Q13113** | **PDZK1-interacting protein 1** | **1.913867183** | **0.243716275** |
| **Q13162** | **Peroxiredoxin-4** | **0.232270665** | **0.417400506** |
| **Q13217** | **DnaJ homolog subfamily C member 3** | **1.0875909** | **0.852237812** |
| **Q13228** | **Methanethiol oxidase** | **0.986002475** | **0.953268983** |
| **Q13232** | **Nucleoside diphosphate kinase 3** | **6.668004964** | **0.34142775** |
| **Q13277** | **Syntaxin-3** | **0.995481232** | **0.977236944** |
| **Q13410** | **Butyrophilin subfamily 1 member A1** | **0.91803349** | **0.582017025** |
| **Q14240** | **Eukaryotic initiation factor 4A-II** | **0.876153242** | **0.430940762** |
| **Q14376** | **UDP-glucose 4-epimerase** | **0.333246304** | **0.223743904** |
| **Q14444** | **Caprin-1** | **0.570918706** | **0.034254317** |
| **Q14512** | **Fibroblast growth factor-binding protein 1** | **0.83386943** | **0.506455069** |
| **Q15084** | **Protein disulfide-isomerase A6** | **0.927756184** | **0.906618626** |
| **Q15833** | **Syntaxin-binding protein 2** | **0.703413301** | **0.283987059** |
| **Q16651** | **Prostasin** | **4.841913651** | **0.407891052** |
| **Q2QD09** | **Triosephosphate isomerase (Fragment)** | **2.343639474** | **0.145248363** |
| **Q4LE33** | **TNC variant protein (Fragment)** | **0.440177688** | **0.364439051** |
| **Q4LE36** | **ATP-citrate (pro-S-)-lyase (Fragment)** | **0.975270966** | **0.866664482** |
| **Q4W4Y1** | **Dopamine receptor interacting protein 4** | **0.842054477** | **0.768266624** |
| **Q53F37** | **SAR1a gene homolog 2 variant (Fragment)** | **0.917631745** | **0.907278561** |
| **Q53FR4** | **Vacuolar protein sorting 35 variant (Fragment)** | **0.803211798** | **0.549168781** |
| **Q53G35** | **Phosphoglycerate mutase (Fragment)** | **1.861422306** | **0.229836929** |
| **Q53GF9** | **Full-length cDNA 5-PRIME end of clone CS0DF013YM24 of Fetal brain of Homo sapiens (Human) variant (Fragment)** | **0.401186687** | **0.286708184** |
| **Q53HU8** | **Vimentin (Fragment)** | **1.314817479** | **0.523419567** |
| **Q53HV2** | **T-complex protein 1 subunit eta (Fragment)** | **1.678485458** | **0.081318047** |
| **Q59E93** | **Aminopeptidase (Fragment)** | **0.623000212** | **0.223590303** |
| **Q59EG8** | **26S proteasome non-ATPase regulatory subunit 2 (Fragment)** | **1.674326083** | **0.226833125** |
| **Q59EJ3** | **Heat shock 70kDa protein 1A variant (Fragment)** | **0.559644053** | **0.029217891** |
| **Q59EN5** | **Prosaposin variant (Fragment)** | **0.773992012** | **0.340542218** |
| **Q59ER5** | **WD repeat-containing protein 1 isoform 1 variant (Fragment)** | **1.531287899** | **0.349435447** |
| **Q59ET0** | **1,4-alpha-glucan branching enzyme (Fragment)** | **1.103077522** | **0.888885003** |
| **Q59F99** | **Staufen isoform b variant (Fragment)** | **1.22405921** | **0.653329524** |
| **Q59FF0** | **100 kDa coactivator (Fragment)** | **1.574376421** | **0.142262074** |
| **Q59G10** | **Formyltetrahydrofolate dehydrogenase (Fragment)** | **1.196957182** | **0.715467075** |
| **Q59G75** | **Isoleucyl-tRNA synthetase (Fragment)** | **0.62081602** | **0.165094997** |
| **Q59GK9** | **60S ribosomal protein L21 (Fragment)** | **1.237858897** | **0.461448526** |
| **Q59GM9** | **Alpha-1,4 glucan phosphorylase (Fragment)** | **0.304118511** | **0.058573355** |
| **Q59GR8** | **TPM1 protein variant (Fragment)** | **1.057225094** | **0.887928611** |
| **Q59GY2** | **60S ribosomal protein L4 (Fragment)** | **0.506617263** | **0.065120524** |
| **Q59H77** | **T-complex protein 1 subunit gamma (Fragment)** | **1.231512278** | **0.439936444** |
| **Q5EC54** | **Heterogeneous nuclear ribonucleoprotein K** | **1.181361658** | **0.401081237** |
| **Q5JR94** | **40S ribosomal protein S8** | **1.464192933** | **0.425178962** |
| **Q5JWF2** | **Guanine nucleotide-binding protein G(s) subunit alpha isoforms XLas** | **1.01727006** | **0.926316913** |
| **Q68CN4** | **Uncharacterized protein** | **0.600342507** | **0.209198218** |
| **Q6FG99** | **RPLP1 protein** | **1.716638411** | **0.010577782** |
| **Q6GMV8** | **Uncharacterized protein** | **0.807611227** | **0.396960706** |
| **Q6IAT9** | **Proteasome subunit beta** | **1.392905521** | **0.123266242** |
| **Q6IB11** | **PGRMC1 protein** | **0.520485043** | **0.165937584** |
| **Q6IPH7** | **60S ribosomal protein L14** | **0.583909794** | **0.08654943** |
| **Q6N089** | **Uncharacterized protein** | **0.781520333** | **0.184099021** |
| **Q6NS36** | **Ferritin (Fragment)** | **0.728051968** | **0.396303134** |
| **Q6NS95** | **IGL@ protein** | **0.712367157** | **0.357017214** |
| **Q6P5S8** | **IGK@ protein** | **0.53215358** | **0.00390455** |
| **Q6PCB0** | **von Willebrand factor A domain-containing protein 1** | **0.904696904** | **0.53894154** |
| **Q6U2E7** | **C4B1 (Fragment)** | **0.80727078** | **0.497344679** |
| **Q6UWW8** | **Carboxylesterase 3** | **3.234025421** | **0.381318424** |
| **Q6UXB2** | **C-X-C motif chemokine 17** | **0.365163057** | **0.408222949** |
| **Q6WN34** | **Chordin-like protein 2** | **0.876203491** | **0.279457894** |
| **Q6ZP37** | **cDNA FLJ26554 fis, clone LNF01773, highly similar to Galactokinase** | **0.518554962** | **0.026725283** |
| **Q6ZVX0** | **cDNA FLJ41981 fis, clone SMINT2011888, highly similar to Protein Tro alpha1 H,myeloma** | **0.317362451** | **0.169366598** |
| **Q6ZW64** | **cDNA FLJ41552 fis, clone COLON2004478, highly similar to Protein Tro alpha1 H,myeloma** | **0.674173686** | **0.117304721** |
| **Q76LA1** | **CSTB protein** | **0.624758756** | **0.485058035** |
| **Q86X29** | **Lipolysis-stimulated lipoprotein receptor** | **1.348518406** | **0.435284353** |
| **Q8IWU5** | **Extracellular sulfatase Sulf-2** | **1.175930138** | **0.276407875** |
| **Q8N474** | **Secreted frizzled-related protein 1** | **0.586717692** | **0.077905712** |
| **Q8N5P9** | **Cell death activator CIDE-A variant 2** | **2.070213541** | **0.512421924** |
| **Q8NBJ4** | **Golgi membrane protein 1** | **0.937283933** | **0.584494484** |
| **Q8NBS9** | **Thioredoxin domain-containing protein 5** | **0.71678811** | **0.762246922** |
| **Q8WVX7** | **40S ribosomal protein S19 (Fragment)** | **1.594590087** | **0.288445047** |
| **Q96IJ6** | **Mannose-1-phosphate guanyltransferase alpha** | **1.36110899** | **0.206423876** |
| **Q96JD0** | **Amyloid lambda 6 light chain variable region SAR (Fragment)** | **1.396527122** | **0.631851475** |
| **Q96KP4** | **Cytosolic non-specific dipeptidase** | **1.453767074** | **0.409577206** |
| **Q96TA1** | **Protein Niban 2** | **0.509139382** | **0.082038182** |
| **Q99541** | **Perilipin-2** | **2.212508336** | **0.405188488** |
| **Q99584** | **Protein S100-A13** | **0.764999249** | **0.490959532** |
| **Q99727** | **Metalloproteinase inhibitor 4** | **1.154923008** | **0.742682447** |
| **Q9BQ13** | **BTB/POZ domain-containing protein KCTD14** | **1.382565495** | **0.576758396** |
| **Q9BRK5** | **45 kDa calcium-binding protein** | **2.568689649** | **0.15562446** |
| **Q9BY76** | **Angiopoietin-related protein 4** | **1.665784471** | **0.179923055** |
| **Q9BYK1** | **40S ribosomal protein S21** | **1.229480205** | **0.749309614** |
| **Q9H173** | **Nucleotide exchange factor SIL1** | **0.773610702** | **0.15522316** |
| **Q9H444** | **Charged multivesicular body protein 4b** | **0.513338622** | **0.069640607** |
| **Q9H772** | **Gremlin-2** | **0.505982818** | **0.117890767** |
| **Q9HB40** | **Retinoid-inducible serine carboxypeptidase** | **0.830759819** | **0.447540412** |
| **Q9HBB3** | **60S ribosomal protein L6** | **1.206972056** | **0.644063895** |
| **Q9HDC9** | **Adipocyte plasma membrane-associated protein** | **0.790782387** | **0.649264885** |
| **Q9NP72** | **Ras-related protein Rab-18** | **0.989472348** | **0.972264357** |
| **Q9NR19** | **Acetyl-coenzyme A synthetase, cytoplasmic** | **0.002301897** | **0.116936639** |
| **Q9NV23** | **S-acyl fatty acid synthase thioesterase, medium chain** | **0.93245478** | **0.777238867** |
| **Q9UII7** | **Cadherin-1** | **0.7702941** | **0.605986561** |
| **Q9UVB1** | **Tubulin beta chain (Fragment)** | **1.08383308** | **0.848346431** |
| **Q9Y2J8** | **Protein-arginine deiminase type-2** | **1.243134139** | **0.334795944** |
| **Q9Y2S2** | **Lambda-crystallin homolog** | **0.923561995** | **0.832088814** |
| **S6BGD4** | **IgG H chain** | **0.809146485** | **0.208226758** |
| **V5YQL4** | **Adenosylhomocysteinase** | **1.13189953** | **0.709848083** |
| **V9H1C1** | **Macrophage-capping protein (Fragment)** | **0.782870277** | **0.438473643** |
| **V9HVY3** | **Protein disulfide-isomerase** | **0.753007693** | **0.82083479** |
| **V9HW01** | **Epididymis secretory protein Li 310** | **2.006895178** | **0.134511707** |
| **V9HW22** | **Epididymis luminal protein 33** | **0.913948456** | **0.599764302** |
| **V9HW88** | **Calreticulin** | **0.925199012** | **0.817266136** |
| **V9HW96** | **CCT-beta** | **0.681669817** | **0.679142323** |
| **V9HWB4** | **78 kDa glucose-regulated protein** | **0.83535542** | **0.65238536** |
| **V9HWB7** | **Citrate hydro-lyase** | **2.03043898** | **0.158413626** |
| **V9HWF4** | **Phosphoglycerate kinase** | **1.888156957** | **0.12872677** |
| **V9HWF6** | **Alpha-1-acid glycoprotein** | **0.707999668** | **0.082714466** |
| **V9HWI0** | **Alcohol dehydrogenase [NADP(+)]** | **1.634969933** | **0.429076379** |
| **W5RWE1** | **Beta-casein** | **1.022397092** | **0.552791775** |
| **W8QEY1** | **Lactotransferrin** | **0.698818972** | **0.292273191** |
| **X5CMJ9** | **Proteasome subunit beta** | **1.021579735** | **0.942634699** |
| **X5D2M8** | **Major vault protein (Fragment)** | **0.88195675** | **0.714010899** |
